# Supplementary material for: Sexual behaviour and incidence of sexually transmitted infections among men who have sex with men (MSM) using daily and event-driven pre-exposure prophylaxis (PrEP): Four-year follow-up of the Amsterdam PrEP (AMPrEP) demonstration project cohort
Source: PLoS Med. 2024 May 8;21(5):e1004328. doi: 10.1371/journal.pmed.1004328 (PMC11111007; doi:10.1371/journal.pmed.1004328)
Supplement: S1 Table — (DOCX) [file pmed.1004328.s001.docx]

**S1 Table.** Sexual behaviour^a^ among 367 AMPrEP participants using daily versus event-driven PrEP over four years in Amsterdam, The Netherlands, 2015-20

|  | Total | |  | Daily | |  | Event-driven | |  |
| --- | --- | --- | --- | --- | --- | --- | --- | --- | --- |
|  | No. visits | Median (IQR) |  | No. visits | Median (IQR) |  | No. visits | Median (IQR) | p-value^b^ |
| No. of sexual partners |  |  |  |  |  |  |  |  |  |
| Overall | 5,105 | 13 (6-26) |  | 3,787 | 15 (7-30) |  | 1,318 | 8 (3-18) | <0.0001 |
| Year 1 | 1,736 | 15 (8-30) |  | 1,290 | 17 (9-32) |  | 446 | 10 (5-21) | <0.0001 |
| Year 2 | 1,269 | 15 (6-27) |  | 942 | 15 (7-30) |  | 327 | 8 (3-16) | <0.0001 |
| Year 3 | 1,168 | 12 (6-25) |  | 859 | 15 (7-26) |  | 309 | 8 (3-15) | <0.0001 |
| Year 4 | 932 | 11 (5-24) |  | 696 | 14 (6-26) |  | 236 | 5 (2-13) | <0.0001 |
| No. of anal sex acts |  |  |  |  |  |  |  |  |  |
| Overall | 5,108 | 18 (9-34) |  | 3,787 | 20 (10-36) |  | 1,321 | 12 (5-25) | <0.0001 |
| Year 1 | 1,738 | 20 (10-35) |  | 1,292 | 22 (12-39) |  | 446 | 14 (6-30) | <0.0001 |
| Year 2 | 1,217 | 19 (9-32) |  | 943 | 21 (11-36) |  | 328 | 12 (4-23) | <0.0001 |
| Year 3 | 1,172 | 16 (8-32) |  | 861 | 19 (10-35) |  | 311 | 11 (5-25) | <0.0001 |
| Year 4 | 931 | 16 (7-30) |  | 695 | 20 (10-33) |  | 236 | 8 (3-20) | <0.0001 |
| No. of CAS acts with casual partners | |  |  |  |  |  |  |  |  |
| Overall | 5,108 | 10 (3-20.5) |  | 3,787 | 11 (4-23) |  | 1,321 | 5 (1-13) | <0.0001 |
| Year 1 | 1,734 | 9 (3-20) |  | 1,288 | 10 (4-22) |  | 446 | 5 (2-13) | <0.0001 |
| Year 2 | 1,269 | 10 (3-22) |  | 941 | 12 (5-25) |  | 328 | 5 (1-13) | <0.0001 |
| Year 3 | 1,173 | 10 (3-21) |  | 862 | 11 (5-24) |  | 311 | 5 (1-15) | <0.0001 |
| Year 4 | 932 | 10 (3-20.5) |  | 696 | 12 (4-23) |  | 236 | 4 (1-10) | <0.0001 |

Abbreviations: AMPrEP, Amsterdam PrEP demonstration project; CAS: condomless anal sex; IQR: interquartile range; PrEP: pre-exposure prophylaxis.

^a^All self-reported and referring to the past 3 months.

^b^p-values were based on the rank sum test.
