# Supplementary material for: Sexual behaviour and incidence of sexually transmitted infections among men who have sex with men (MSM) using daily and event-driven pre-exposure prophylaxis (PrEP): Four-year follow-up of the Amsterdam PrEP (AMPrEP) demonstration project cohort
Source: PLoS Med. 2024 May 8;21(5):e1004328. doi: 10.1371/journal.pmed.1004328 (PMC11111007; doi:10.1371/journal.pmed.1004328)
Supplement: S2 Table — (DOCX) [file pmed.1004328.s002.docx]

| **S2 Table.** Four-year outcomes of sexual behaviour per year since PrEP initiation among 367 AMPrEP participants, Amsterdam, The Netherlands, 2015-20 | | | | | | | | |
| --- | --- | --- | --- | --- | --- | --- | --- | --- |
|  |  | Crude | | |  | Adjusted^a^ | | |
|  | No. of visits with data | RR [95% CI] | | p-value^c^ |  | aRR [95% CI] | | p-value^c^ |
| Number of sexual partners^b^ |  |  |  |  |  |  |  |  |
| Overall | 5,105 | 0.88 | [0.86-0.91] | **<0.0001** |  | 0.89 | [0.86-0.91] | **<0.0001** |
| Daily | 3,787 | 0.90 | [0.87-0.93] | **<0.0001** |  | 0.90 | [0.87-0.93] | **<0.0001** |
| Event-driven | 1,318 | 0.86 | [0.81-0.91] | **<0.0001** |  | 0.86 | [0.81-0.92] | **<0.0001** |
| Number of anal sex acts^b^ |  |  |  |  |  |  |  |  |
| Overall | 5,112 | 0.92 | [0.89-0.94] | **<0.0001** |  | 0.92 | [0.89-0.95] | **<0.0001** |
| Daily | 3,791 | 0.92 | [0.89-0.94] | **<0.0001** |  | 0.94 | [0.91-0.97] | **<0.0001** |
| Event-driven | 1,321 | 0.93 | [0.90-0.96] | **<0.0001** |  | 0.88 | [0.83-0.93] | **<0.0001** |
| Number of CAS acts with casual partners^b^ | |  |  |  |  |  |  |  |
| Overall | 5,108 | 0.95 | [0.92-0.99] | **0.0079** |  | 0.95 | [0.92-0.99] | **0.013** |
| Daily | 3,787 | 0.98 | [0.94-1.02] | 0.25 |  | 0.97 | [0.93-1.01] | 0.21 |
| Event-driven | 1,321 | 0.92 | [0.85-0.99] | 0.032 |  | 0.92 | [0.85-0.99] | **0.032** |
| Abbreviations: AMPrEP: Amsterdam PrEP demonstration project; CAS: condomless anal sex; CI: confidence interval;  PrEP: pre-exposure prophylaxis; (a)RR: (adjusted) rate ratio. | | | | | | | | |
| ^a^Adjusted for age at enrolment modelled as cubic splines with four knots, and including a random intercept and random slope at the participant-level. ^b^In the past 3 months  ^c^p-values based on the Wald test | | | | | | | | |
