# Supplementary material for: Sexual behaviour and incidence of sexually transmitted infections among men who have sex with men (MSM) using daily and event-driven pre-exposure prophylaxis (PrEP): Four-year follow-up of the Amsterdam PrEP (AMPrEP) demonstration project cohort
Source: PLoS Med. 2024 May 8;21(5):e1004328. doi: 10.1371/journal.pmed.1004328 (PMC11111007; doi:10.1371/journal.pmed.1004328)
Supplement: S3 Table — (DOCX) [file pmed.1004328.s003.docx]

| **S3 Table.** Four-year outcomes of incidence of STIs overall and by PrEP regimen, among 367 AMPrEP participants, Amsterdam, the Netherlands, 2015-20 | | | | | | | | | | | | | | | | | | |
| --- | --- | --- | --- | --- | --- | --- | --- | --- | --- | --- | --- | --- | --- | --- | --- | --- | --- | --- |
|  | **Total** | | | | |  | **Daily PrEP** | | | | |  | **Event-driven PrEP** | | | | |  |
|  | No. of participants with ≥1 positive test | No. of positive tests | PY | IR per 100PY [95% CI] | |  | No. of participants with ≥1 positive test | No. of positive tests | PY | IR per 100PY [95% CI] | |  | No. of participants with ≥1 positive test | No. of positive tests | PY | IR per 100PY [95% CI] | | p-value^a^ |
| **Any STI**^b^ | 289 | 1092 | 1258 | 86.8 | [81.8-92.1] |  | 246 | 891 | 914 | 97.4 | [91.3-104] |  | 89 | 201 | 344 | 58.5 | [50.9-67.2] | **<0.0001** |
| **Any anal STI**^c^ | 241 | 758 | 1258 | 60.3 | [56.1-64.7] |  | 208 | 630 | 914 | 68.9 | [63.7-74.5] |  | 66 | 128 | 344 | 37.2 | [31.3-44.3] | **<0.0001** |
| **Chlamydia** |  |  |  |  |  |  |  |  |  |  |  |  |  |  |  |  |  |  |
| Any chlamydia | 227 | 524 | 1258 | 41.7 | [38.2-45.4] |  | 197 | 432 | 914 | 47.2 | [43.0-51.9] |  | 54 | 92 | 344 | 26.8 | [21.8-32.8] | **<0.0001** |
| Anal chlamydia | 197 | 423 | 1258 | 33.6 | [30.6-37.0] |  | 171 | 351 | 914 | 38.4 | [34.6-42.6] |  | 46 | 72 | 344 | 20.9 | [16.6-26.4] | **<0.0001** |
| Urogenital chlamydia | 95 | 132 | 1258 | 10.5 | [8.8-12.4] |  | 78 | 104 | 914 | 11.4 | [9.4-13.8] |  | 20 | 28 | 344 | 8.1 | [5.6-11.8] | 0.13 |
| Pharyngeal chlamydia | 34 | 46 | 1258 | 3.7 | [2.7-4.9] |  | 26 | 38 | 914 | 4.2 | [3.0-5.7] |  | 8 | 8 | 344 | 2.3 | [1.2-4.7] | 0.28 |
| LGV | 59 | 69 | 1258 | 5.5 | [4.3-6.9] |  | 53 | 60 | 914 | 6.6 | [5.1-8.5] |  | 8 | 9 | 344 | 2.6 | [1.4-5.0] | **0.0040** |
| **Gonorrhoea** |  |  |  |  |  |  |  |  |  |  |  |  |  |  |  |  |  |  |
| Any gonorrhoea | 234 | 615 | 1258 | 48.9 | [45.2-52.9] |  | 195 | 505 | 914 | 55.2 | [50.6-60.3] |  | 61 | 110 | 344 | 32.0 | [26.6-38.6] | **<0.0001** |
| Anal gonorrhoea | 177 | 432 | 1258 | 34.3 | [31.2-37.7] |  | 150 | 359 | 914 | 39.3 | [35.4-43.5] |  | 43 | 73 | 344 | 21.2 | [16.9-26.7] | **<0.0001** |
| Urogenital gonorrhoea | 69 | 121 | 1258 | 9.6 | [8.0-11.5] |  | 59 | 106 | 914 | 11.6 | [9.6-14.0] |  | 13 | 15 | 344 | 4.4 | [2.6-7.2] | **0.0001** |
| Pharyngeal gonorrhoea | 157 | 282 | 1258 | 22.4 | [19.9-25.2] |  | 128 | 223 | 914 | 24.4 | [21.4-27.8] |  | 44 | 59 | 344 | 17.2 | [13.3-22.2] | **0.016** |
| **Infectious syphilis**^d^ | 111 | 140 | 1258 | 11.1 | [9.4-13.1] |  | 86 | 108 | 914 | 11.8 | [9.8-14.3] |  | 30 | 32 | 344 | 9.3 | [6.6-13.2] | 0.27 |
| **HIV** | 2 | 2 | 1258 | 0.2 | [0.0-0.6] |  | 2 | 2 | 914 | 0.2 | [0.1-0.9] |  | 0 | 0 | 344 | 0.0 | [0.0-1.1] | 0.53 |
| **HCV** |  |  |  |  |  |  |  |  |  |  |  |  |  |  |  |  |  |  |
| Any new infection | 15^g^ | 17 | 1186 | 1.4 | [0.9-2.3] |  | 14 | 15 | 870 | 1.7 | [1.0-2.9] |  | 2 | 2 | 315 | 0.6 | [0.2-2.5] | 0.17 |
| First infection^e^ | 10 | 10 | 1126 | 0.9 | [0.5-1.7] |  | 9 | 9 | 837 | 1.1 | [0.6-2.1] |  | 1 | 1 | 289 | 0.3 | [0.0-2.5] | 0.28 |
| Re-infection^f^ | 6 | 7 | 60 | 11.7 | [5.6-25] |  | 5 | 7 | 33 | 17.9 | [8.1-40] |  | 1 | 1 | 26 | 3.8 | [0.5-27.0] | 0.12 |
| Abbreviations: AMPrEP: Amsterdam PrEP demonstration project; CI: confidence interval; HCV: hepatitis C virus; HIV: human immunodeficiency virus; IR: incidence rate;  LGV: lymphogranuloma venereum; PrEP: pre-exposure prophylaxis; PY: person-years; STI: sexually transmitted infection. | | | | | | | | | | | | | | | | | | |
| ^a^Two-sided p-values for the crude incidence rate difference between daily and event-driven PrEP users were based on the Z-test | | | | | | | | | | | | | | | | | | |
| ^b^Any STI: chlamydia (any location), gonorrhoea (any location), infectious syphilis (stage 1, 2 and recent latent infection) | | | | | | | | | | | | | | | | | | |
| ^c^Any anal STI: anal chlamydia or anal gonorrhea | | | | | | | | | | | | | | | | | | |
| ^d^Syphilis stage 1, stage 2 and recent latent infection | | | | | | | | | | | | | | | | | | |
| ^e^First infection: based on RNA positivity, no (known) history of HCV, and prior negative HCV antibodies | | | | | | | | | | | | | | | | | | |
| ^f^Re-infection: based on ribonucleic acid (RNA) positivity and known history of HCV or prior positive HCV antibodies | | | | | | | | | | | | | | | | | | |
| ^g^One individual had a first HCV infection and HCV re-infection during follow-up | | | | | | | |  |  |  |  |  |  |  |  |  |  |  |
