# Supplementary material for: Sexual behaviour and incidence of sexually transmitted infections among men who have sex with men (MSM) using daily and event-driven pre-exposure prophylaxis (PrEP): Four-year follow-up of the Amsterdam PrEP (AMPrEP) demonstration project cohort
Source: PLoS Med. 2024 May 8;21(5):e1004328. doi: 10.1371/journal.pmed.1004328 (PMC11111007; doi:10.1371/journal.pmed.1004328)
Supplement: S4 Table — (DOCX) [file pmed.1004328.s004.docx]

| **S4 Table.** Four-year outcomes of incidence of STIs overall and by PrEP regimen, among 142 AMPrEP participants who ever switched their PrEP regimen, Amsterdam, the Netherlands, 2015-20 | | | | | | | | | | | | | | | | | | |
| --- | --- | --- | --- | --- | --- | --- | --- | --- | --- | --- | --- | --- | --- | --- | --- | --- | --- | --- |
|  | **Total** | | | | |  | **Daily PrEP** | | | | |  | **Event-driven PrEP** | | | | |  |
|  | No. of participants with ≥1 positive test | No. of positive tests | PY | IR per 100PY [95% CI] | |  | No. of participants with ≥1 positive test | No. of positive tests | PY | IR per 100PY [95% CI] | |  | No. of participants with ≥1 positive test | No. of positive tests | PY | IR per 100PY [95% CI] | | p-value^a^ |
| **Any STI**^b^ | 108 | 405 | 490 | 82.5 | [74.9-91.0] |  | 93 | 291 | 302 | 96.5 | [86.0-108.3] |  | 61 | 114 | 189 | 60.4 | [50.2-72.5] | **<0.0001** |
| **Any anal STI**^c^ | 87 | 270 | 490 | 55.1 | [48.9-62.0] |  | 76 | 193 | 302 | 64.0 | [55.6-73.7] |  | 43 | 77 | 189 | 40.8 | [32.6-51.0] | **0.0006** |
| **Chlamydia** |  |  |  |  |  |  |  |  |  |  |  |  |  |  |  |  |  |  |
| Any chlamydia | 77 | 180 | 490 | 36.7 | [31.7-42.5] |  | 68 | 133 | 302 | 44.1 | [37.2-52.3] |  | 32 | 47 | 189 | 24.9 | [18.7-33.1] | **0.0005** |
| Anal chlamydia | 66 | 139 | 490 | 28.3 | [24.0-33.5] |  | 58 | 99 | 302 | 32.8 | [27.0-40.0] |  | 26 | 40 | 189 | 21.2 | [15.5-28.9] | **0.017** |
| Urogenital chlamydia | 33 | 47 | 490 | 9.6 | [7.2-12.8] |  | 26 | 36 | 302 | 11.9 | [8.6-16.6] |  | 10 | 11 | 189 | 5.8 | [3.2-10.5] | **0.031** |
| Pharyngeal chlamydia | 13 | 19 | 490 | 3.9 | [2.5-6.1] |  | 10 | 15 | 302 | 5.0 | [3.0-8.3] |  | 3 | 4 | 189 | 2.1 | [0.8-5.6] | 0.12 |
| LGV | 14 | 21 | 490 | 4.3 | [2.8-6.6] |  | 13 | 17 | 302 | 5.6 | [3.5-9.1] |  | 3 | 4 | 189 | 2.1 | [0.8-5.6] | 0.065 |
| **Gonorrhoea** |  |  |  |  |  |  |  |  |  |  |  |  |  |  |  |  |  |  |
| Any gonorrhoea | 95 | 230 | 490 | 46.9 | [41.2-53.4] |  | 73 | 161 | 302 | 53.4 | [45.8-62.3] |  | 44 | 69 | 189 | 36.5 | [28.9-46.3] | **0.0072** |
| Anal gonorrhoea | 70 | 154 | 490 | 31.4 | [26.8-36.8] |  | 53 | 108 | 302 | 35.8 | [29.7-43.3] |  | 32 | 46 | 189 | 24.4 | [18.2-32.5] | **0.026** |
| Urogenital gonorrhoea | 28 | 52 | 490 | 10.6 | [8.1-13.9] |  | 23 | 43 | 302 | 14.3 | [10.6-19.2] |  | 8 | 9 | 189 | 4.8 | [2.5-9.2] | **0.0011** |
| Pharyngeal gonorrhoea | 67 | 122 | 490 | 24.9 | [20.8-29.7] |  | 50 | 83 | 302 | 27.5 | [22.2-34.1] |  | 32 | 39 | 189 | 20.6 | [15.1-28.3] | 0.14 |
| **Infectious syphilis**^d^ | 39 | 53 | 490 | 10.8 | [8.3-14.1] |  | 28 | 37 | 302 | 12.3 | [8.9-16.9] |  | 15 | 16 | 189 | 8.5 | [5.2-13.8] | 0.21 |
| **HIV** | 0 | 0 | - | - | - |  | 0 | 0 | - | - | - |  | 0 | 0 | - | - | - | - |
| **HCV** |  |  |  |  |  |  |  |  |  |  |  |  |  |  |  |  |  |  |
| Any new infection | 7 | 8 | 452 | 1.8 | [0.9-3.5] |  | 6 | 6 | 283 | 2.1 | [1.0-4.7] |  | 2 | 2 | 169 | 1.2 | [0.3-4.7] | 0.50 |
| First infection^e^ | 5 | 5 | 417 | 1.2 | [0.5-2.9] |  | 4 | 4 | 266 | 1.5 | [0.6-4.0] |  | 1 | 1 | 151 | 0.7 | [0.1-4.7] | 0.51 |
| Re-infection^f^ | 3 | 3 | 35 | 8.5 | [2.7-26.4] |  | 2 | 2 | 18 | 11.3 | [2.8-45.2] |  | 1 | 1 | 18 | 5.7 | [0.8-40.5] | 0.59 |
| Abbreviations: AMPrEP: Amsterdam PrEP demonstration project; CI: confidence interval; HCV: hepatitis C virus; HIV: human immunodeficiency virus; IR: incidence rate;  LGV: lymphogranuloma venereum; PrEP: pre-exposure prophylaxis; PY: person-years; STI: sexually transmitted infection. | | | | | | | | | | | | | | | | | | |
| ^a^Two- sided p-values for the crude incidence rate difference between daily and event-driven PrEP users were based on the Z-test | | | | | | | | | | | | | | | | | | |
| ^b^Any STI: chlamydia (any location), gonorrhoea (any location), infectious syphilis (stage 1, 2 and recent latent infection) | | | | | | | | | | | | | | | | | | |
| ^c^Any anal STI: anal chlamydia or anal gonorrhoea | | | | | | | | | | | | | | | | | | |
| ^d^Syphilis stage 1, stage 2 and recent latent infection | | | | | | | | | | | | | | | | | | |
| ^e^First infection: based on ribonucleic acid (RNA) positivity, no (known) history of HCV, and prior negative HCV antibodies | | | | | | | | | | | | | | | | | | |
| ^f^Re-infection: based on RNA positivity and known history of HCV or prior positive HCV antibodies | | | | | | | | | | | | | | | | | | |
| ^g^One individual had a first HCV infection and HCV re-infection during follow-up | | | | | | | |  |  |  |  |  |  |  |  |  |  |  |
