# Supplementary material for: Sexual behaviour and incidence of sexually transmitted infections among men who have sex with men (MSM) using daily and event-driven pre-exposure prophylaxis (PrEP): Four-year follow-up of the Amsterdam PrEP (AMPrEP) demonstration project cohort
Source: PLoS Med. 2024 May 8;21(5):e1004328. doi: 10.1371/journal.pmed.1004328 (PMC11111007; doi:10.1371/journal.pmed.1004328)
Supplement: S5 Table — (DOCX) [file pmed.1004328.s005.docx]

| **S5 Table.** Incidence rate ratio for STIs per additional year on PrEP over four years of PrEP use among 367 AMPrEP participants, Amsterdam, The Netherlands, 2015-20   \|  \| Total \| \| \| \| \| \| \| \|  \| \| Daily PrEP \| \| \| \| \| \| \| \|  \| \| Event-driven PrEP \| \| \| \| \| \| \| \| \| \| --- \| --- \| --- \| --- \| --- \| --- \| --- \| --- \| --- \| --- \| --- \| --- \| --- \| --- \| --- \| --- \| --- \| --- \| --- \| --- \| --- \| --- \| --- \| --- \| --- \| --- \| --- \| --- \| --- \| --- \| \|  \| IR/ 100 PY  [95% CI] \| Crude IRR  [95% CI] \| \| aIRR^c^ [95% CI] \| \| \| p-value^d^ \| \|  \| \| IR/ 10oPY  [95% CI] \| \| aIRR^c^ [95% CI] \| \| \| \| p-value^d^ \| \|  \| \| IR/ 100 PY  [95% CI] \| aIRR^c^ [95% CI] \| \| \| \| \| p-value^d^ \| \| \| \| **Any STI**^a^ \|  \|  \|  \| \|  \|  \| \|  \| \|  \| \|  \| \| \|  \|  \| \| \|  \| \|  \| \| \|  \| \|  \|  \| \| \| \| \| 0-12 months on PrEP \| 93.1 [83.7-103.7] \| Ref. \|  \| \| Ref. \|  \| \|  \| \| 106.9 [95.1-120.1] \| \| \| \| Ref. \|  \| \| \|  \| \| 54.8 [41.7-71.9] \| \| \| Ref. \| \|  \| \| \|  \| \| \| \| 12-24 months on PrEP \| 84.8 [75.5-95.3] \| 0.91 \| [0.77-1.07] \| \| 0.77 \| [0.65-0.91] \| \| **0.0019** \| \| 93.8 [82.5-106.7] \| \| \| \| 0.78 \| [0.65-0.94] \| \| \| **0.0075** \| \| 59.5 [54.3-78.1] \| \| \| 0.76 \| \| [0.50-1.13] \| \| \| 0.18 \| \| \| \| 24-36 months on PrEP \| 83.4 [73.9-94.2] \| 0.90 \| [0.76-1.06] \| \| 0.78 \| [0.66-0.92] \| \| **0.0034** \| \| 91.7 [80.0-105.1] \| \| \| \| 0.77 \| [0.63-0.92] \| \| \| **0.0054** \| \| 62.0 [47.5-81.0] \| \| \| 0.94 \| \| [0.63-1.40] \| \| \| 0.77 \| \| \| \| 36-48 months on PrEP \| 84.5 [73.9-96.7] \| 0.91 \| [0.76-1.06] \| \| 0.89 \| [0.75-1.06] \| \| 0.201 \| \| 95.6 [82.3-111.1] \| \| \| \| 0.89 \| [0.73-1.08] \| \| \| 0.23 \| \| 57.8 [42.9-78.0] \| \| \| 1.04 \| \| [0.68-1.58] \| \| \| 0.85 \| \| \| \| **Any anal STI**^b^ \|  \|  \|  \| \|  \|  \| \|  \| \|  \| \| \| \|  \|  \| \| \|  \| \|  \| \| \|  \| \|  \| \| \|  \| \| \| \| 0-12 months on PrEP \| 62.0 [54.4-70.7] \| Ref. \|  \| \| Ref. \|  \| \|  \| \| 73.7 [64.0-84.8] \| \| \| \| Ref. \|  \| \| \|  \| \| 29.5 [20.4-42.7] \| \| \| Ref. \| \|  \| \| \|  \| \| \| \| 12-24 months on PrEP \| 55.5 [48.0-64.0] \| 0.89 \| [0.73-1.09] \| \| 0.76 \| [0.62-0.94] \| \| **0.010** \| \| 63.4 [54.2-74.1] \| \| \| \| 0.78 \| [0.62-0.97] \| \| \| **0.025** \| \| 33.2 [23.1-47.8] \| \| \| 0.77 \| \| [0.44-1.35] \| \| \| 0.37 \| \| \| \| 24-36 months on PrEP \| 63.9 [55.6-73.4] \| 1.03 \| [0.85-1.25] \| \| 0.91 \| [0.75-1.11] \| \| 0.37 \| \| 69.5 [59.4-81.3] \| \| \| \| 0.86 \| [0.69-1.08] \| \| \| 0.19 \| \| 49.4 [36.6-66.6] \| \| \| 1.38 \| \| [0.83-2.30] \| \| \| 0.21 \| \| \| \| 36-48 months on PrEP \| 59.7 [50.9-70.0] \| 0.96 \| [0.78-1.25] \| \| 0.95 \| [0.77-1.17] \| \| 0.60 \| \| 68.8 [57.7-82.1] \| \| \| \| 0.93 \| [0.74-1.18] \| \| \| 0.56 \| \| 37.7 [26.0-54.5] \| \| \| 1.26 \| \| [0.72-2.21] \| \| \| 0.41 \| \| \| \| **Chlamydia** \|  \|  \|  \| \|  \|  \| \|  \| \|  \| \| \| \|  \|  \| \| \|  \| \|  \| \| \|  \| \|  \| \| \|  \| \| \| \| Any chlamydia \|  \|  \|  \| \|  \|  \| \|  \| \|  \| \| \| \|  \|  \| \| \|  \| \|  \| \| \|  \| \|  \| \| \|  \| \| \| \| 0-12 months on PrEP \| 46.4 [39.9-54.0] \| Ref. \|  \| \| Ref. \|  \| \|  \| \| 53.6 [45.5-63.2] \| \| \| \| Ref. \|  \| \| \|  \| \| 26.3 [17.8-39.0] \| \| \| Ref. \| \|  \| \| \|  \| \| \| \| 12-24 months on PrEP \| 37.5 [31.4-44.7] \| 0.81 \| [0.63-1.02] \| \| 0.69 \| [0.54-0.88] \| \| **0.0024** \| \| 41.8 [34.5-50.7] \| \| \| \| 0.71 \| [0.54-0.92] \| \| \| **0.0097** \| \| 25.2 [16.6-38.2] \| \| \| 0.67 \| \| [0.37-1.22] \| \| \| 0.19 \| \| \| \| 24-36 months on PrEP \| 41.7 [35.1-49.5] \| 0.90 \| [0.71-1.14] \| \| 0.78 \| [0.62-0.99] \| \| **0.044** \| \| 46.8 [38.6-56.6] \| \| \| \| 0.79 \| [0.61-1.03] \| \| \| 0.084 \| \| 28.7 [19.4-42.5] \| \| \| 0.89 \| \| [0.50-1.58] \| \| \| 0.69 \| \| \| \| 36-48 months on PrEP \| 40.3 [33.2-48.9] \| 0.87 \| [0.67-1.14] \| \| 0.86 \| [0.67-1.10] \| \| 0.23 \| \| 45.9 [36.9-56.9] \| \| \| \| 0.86 \| [0.65-1.14] \| \| \| 0.29 \| \| 26.9 [17.4-41.7] \| \| \| 1.00 \| \| [0.54-1.86] \| \| \| 0.99 \| \| \| \| Anal chlamydia \|  \|  \|  \| \|  \|  \| \|  \| \|  \| \| \| \|  \|  \| \| \|  \| \|  \| \| \|  \| \|  \| \| \|  \| \| \| \| 0-12 months on PrEP \| 36.1 [30.4-42.9] \| Ref. \|  \| \| Ref. \|  \| \|  \| \| 42.3 [35.2-50.9] \| \| \| \| Ref. \|  \| \| \|  \| \| 19.0 [11.9-30.1] \| \| \| Ref. \| \|  \| \| \|  \| \| \| \| 12-24 months on PrEP \| 28.2 [23.0-34.5] \| 0.78 \| [0.59-1.02] \| \| 0.68 \| [0.52-0.90] \| \| **0.0066** \| \| 31.7 [25.4-39.6] \| \| \| \| 0.70 \| [0.52-0.95] \| \| \| **0.020** \| \| 18.3 [11.2-29.9] \| \| \| 0.65 \| \| [0.32-1.33] \| \| \| 0.24 \| \| \| \| 24-36 months on PrEP \| 35.3 [29.3-42.6] \| 0.98 \| [0.75-1.27] \| \| 0.87 \| [0.67-1.13] \| \| 0.29 \| \| 38.7 [31.4-47.8] \| \| \| \| 0.86 \| [0.64-1.15] \| \| \| 0.31 \| \| 26.4 [17.6-39.8] \| \| \| 1.09 \| \| [0.57-2.08] \| \| \| 0.80 \| \| \| \| 36-48 months on PrEP \| 35.2 [28.6-43.3] \| 0.97 \| [0.73-1.27] \| \| 0.97 \| [0.74-1.27] \| \| 0.81 \| \| 41.4 [33.0-52.0] \| \| \| \| 1.00 \| [0.74-1.34] \| \| \| 0.98 \| \| 20.2 [12.2-33.5] \| \| \| 1.00 \| \| [0.49-2.06] \| \| \| 0.99 \| \| \| \| Urogenital chlamydia \|  \|  \|  \| \|  \|  \| \|  \| \|  \| \| \| \|  \|  \| \| \|  \| \|  \| \| \|  \| \|  \| \| \|  \| \| \| \| 0-12 months on PrEP \| 11.1 [8.2-15.2] \| Ref. \|  \| \| Ref. \|  \| \|  \| \| 12.8 [9.2-18.0] \| \| \| \| Ref. \|  \| \| \|  \| \| 6.3 [2.8-14.1] \| \| \| Ref. \| \|  \| \| \|  \| \| \| \| 12-24 months on PrEP \| 10.5 [7.5-14.6] \| 0.94 \| [0.58-1.52] \| \| 0.76 \| [0.48-1.23] \| \| 0.26 \| \| 10.2 [6.9-15.0] \| \| \| \| 0.66 \| [0.39-1.13] \| \| \| 0.13 \| \| 11.4 [6.2-21.3] \| \| \| 1.34 \| \| [0.46-3.91] \| \| \| 0.59 \| \| \| \| 24-36 months on PrEP \| 12.5 [9.1-17.1] \| 1.13 \| [0.71-1.79] \| \| 0.97 \| [0.61-1.53] \| \| 0.88 \| \| 14.2 [10.1-20.1] \| \| \| \| 0.95 \| [0.57-1.57] \| \| \| 0.83 \| \| 8.0 [3.8-16.9] \| \| \| 1.22 \| \| [0.40-3.76] \| \| \| 0.73 \| \| \| \| 36-48 months on PrEP \| 7.1 [4.5-11.3] \| 0.64 \| [0.35-1.79] \| \| 0.65 \| [0.37-1.13] \| \| 0.13 \| \| 7.3 [4.2-12.5] \| \| \| \| 0.57 \| [0.30-1.08] \| \| \| 0.083 \| \| 6.7 [2.8-16.2] \| \| \| 1.20 \| \| [0.35-4.06] \| \| \| 0.77 \| \| \| \| Pharyngeal chlamydia \|  \|  \|  \| \|  \|  \| \|  \| \|  \| \| \| \|  \|  \| \| \|  \| \|  \| \| \|  \| \|  \| \| \|  \| \| \| \| 0-12 months on PrEP \| 4.4 [2.7-7.3] \| Ref. \|  \| \| Ref. \|  \| \|  \| \| 4.5 [2.6-8.0] \| \| \| \| Ref. \|  \| \| \|  \| \| 4.2 [1.6-11.2] \| \| \| Model did not converge \| \| \| \| \| \| \| 12-24 months on PrEP \| 3.3 [1.8-6.0] \| 0.74 \| [0.31-1.70] \| \| 0.54 \| [0.25-1.20] \| \| 0.13 \| \| 3.2 [1.6-6.5] \| \| \| \| 0.60 \| [0.24-1.53] \| \| \| 0.29 \| \| 3.4 [1.1-10.6] \| \| \|  \| \|  \| \| \|  \| \| \| \| 24-36 months on PrEP \| 4.2 [2.4-7.2] \| 0.94 \| [0.41-2.08] \| \| 0.68 \| [0.32-1.46] \| \| 0.32 \| \| 5.8 [3.4-10.0] \| \| \| \| 1.04 \| [0.93-2.40] \| \| \| 0.93 \| \| 0.0 [0.0-0.0] \| \| \|  \| \|  \| \| \|  \| \| \| \| 36-48 months on PrEP \| 2.4 [1.0-5.3] \| 0.53 \| [0.17-2.08] \| \| 0.51 \| [0.19-1.32] \| \| 0.16 \| \| 2.8 [1.2-6.7] \| \| \| \| 0.60 \| [0.21-1.75] \| \| \| 0.35 \| \| 1.3 [0.2-9.5] \| \| \|  \| \|  \| \| \|  \| \| \|  \| LGV \|  \|  \|  \|  \|  \|  \|  \|  \|  \|  \|  \|  \| \|  \| \|  \|  \| \| \| --- \| --- \| --- \| --- \| --- \| --- \| --- \| --- \| --- \| --- \| --- \| --- \| --- \| --- \| --- \| --- \| --- \| --- \| --- \| \| 0-12 months on PrEP \| 4.7 [2.9-7.6] \| Ref. \|  \| Ref. \|  \|  \| 6.0 [3.7-9.9] \| Ref. \|  \|  \| 1.1 [0.1-7.5] \| \| Ref. \| \|  \| \| \|  \| \| \| \| 12-24 months on PrEP \| 4.2 [2.6-7.1] \| 0.89 \| [0.41-1.91] \| 0.65 \| [0.31-1.34] \| 0.25 \| 4.9 [2.8-8.6] \| 0.66 \| [0.31-1.44] \| 0.30 \| 2.3 [0.6-9.2] \| \| 0.94 \| \| [0.08-11.04] \| \| \| 0.96 \| \| \| \| 24-36 months on PrEP \| 6.1 [3.9-9.6] \| 1.29 \| [0.63-2.64] \| 1.04 \| [0.53-2.03] \| 0.92 \| 8.0 [5.0-12.7] \| 1.13 \| [0.56-2.27] \| 0.74 \| 1.1 [0.2-8.2] \| \| 0.82 \| \| [0.05-13.61] \| \| \| 0.89 \| \| \| \| 36-48 months on PrEP \| 7.5 [4.8-11.8] \| 1.59 \| [0.78-2.64] \| 1.60 \| [0.82-3.11] \| 0.16 \| 7.8 [4.6-13.2] \| 1.32 \| [0.64-2.72] \| 0.46 \| 6.7 [2.8-16.2] \| \| 8.49 \| \| [0.89-80.69] \| \| \| 0.063 \| \| \| \| **Gonorrhoea** \|  \|  \|  \|  \|  \|  \|  \|  \|  \|  \|  \| \|  \| \|  \| \| \|  \| \| \| \| Any gonorrhoea \|  \|  \|  \|  \|  \|  \|  \|  \|  \|  \|  \| \|  \| \|  \| \| \|  \| \| \| \| 0-12 months on PrEP \| 51.7 [44.8-59.7] \| Ref. \|  \| Ref. \|  \|  \| 60.1 [51.4-70.2] \| Ref. \|  \|  \| 28.4 [19.5-41.5] \| \| Ref. \| \|  \| \| \|  \| \| \| \| 12-24 months on PrEP \| 48.0 [41.1-56.0] \| 0.93 \| [0.75-1.15] \| 0.75 \| [0.60-0.94] \| **0.011** \| 54.8 [46.3-64.9] \| 0.78 \| [0.61-0.99] \| **0.037** \| 28.6 [19.3-42.3] \| \| 0.67 \| \| [0.38-1.19] \| \| \| 0.17 \| \| \| \| 24-36 months on PrEP \| 47.5 [40.4-55.8] \| 0.92 \| [0.74-1.15] \| 0.78 \| [0.62-0.98] \| **0.030** \| 51.7 [43.1-62.0] \| 0.73 \| [0.57-0.94] \| **0.015** \| 36.8 [26.0-52.0] \| \| 1.14 \| \| [0.67-1.94] \| \| \| 0.64 \| \| \| \| 36-48 months on PrEP \| 47.8 [40.0-57.1] \| 0.92 \| [0.73-1.15] \| 0.92 \| [0.73-1.16] \| 0.46 \| 53.1 [43.5-65.0] \| 0.88 \| [0.67-1.14] \| 0.32 \| 35.0 [23.8-51.4] \| \| 1.30 \| \| [0.74-2.29] \| \| \| 0.36 \| \| \| \| Anal gonorrhoea \|  \|  \|  \|  \|  \|  \|  \|  \|  \|  \|  \| \|  \| \|  \| \| \|  \| \| \| \| 0-12 months on PrEP \| 35.3 [29.7-42.0] \| Ref. \|  \| Ref. \|  \|  \| 42.3 [35.2-50.9] \| Ref. \|  \|  \| 15.8 [9.5-26.2] \| \| Ref. \| \|  \| \| \|  \| \| \| \| 12-24 months on PrEP \| 32.4 [26.8-39.1] \| 0.92 \| [0.70-1.19] \| 0.74 \| [0.56-0.96] \| **0.026** \| 37.0 [30.9-45.4] \| 0.75 \| [0.56-1.00] \| **0.048** \| 19.5 [12.1-31.3] \| \| 0.78 \| \| [0.37-1.65] \| \| \| 0.52 \| \| \| \| 24-36 months on PrEP \| 36.3 [30.2-43.6] \| 1.03 \| [0.79-1.33] \| 0.88 \| [0.68-1.15] \| 0.36 \| 39.6 [32.2-48.8] \| 0.81 \| [0.60-1.09] \| 0.17 \| 27.6 [18.5-41.1] \| \| 1.63 \| \| [0.82-3.25] \| \| \| 0.17 \| \| \| \| 36-48 months on PrEP \| 33.2 [26.8-41.1] \| 0.94 \| [0.70-1.33] \| 0.92 \| [0.69-1.21] \| 0.54 \| 37.5 [29.5-47.6] \| 0.86 \| [0.63-1.18] \| 0.36 \| 22.9 [14.2-36.8] \| \| 1.67 \| \| [0.80-3.53] \| \| \| 0.17 \| \| \| \| Urogenital gonorrhoea \|  \|  \|  \|  \|  \|  \|  \|  \|  \|  \|  \| \|  \| \|  \| \| \|  \| \| \| \| 0-12 months on PrEP \| 9.5 [6.8-13.2] \| Ref. \|  \| Ref. \|  \|  \| 11.3 [7.9-16.2] \| Ref. \|  \|  \| 4.2 [1.6-11.2] \| \| Ref. \| \|  \| \| \|  \| \| \| \| 12-24 months on PrEP \| 9.0 [6.3-12.9] \| 0.95 \| [0.56-1.60] \| 0.78 \| [0.47-1.31] \| 0.35 \| 10.6 [7.2-15.5] \| 0.78 \| [0.45-1.35] \| 0.37 \| 4.6 [1.7-12.2] \| \| 0.92 \| \| [0.21-4.03] \| \| \| 0.91 \| \| \| \| 24-36 months on PrEP \| 9.0 [6.2-13.0] \| 0.95 \| [0.56-1.62] \| 0.81 \| [0.48-1.37] \| 0.44 \| 10.2 [6.8-15.4] \| 0.72 \| [0.40-1.28] \| 0.26 \| 5.7 [2.4-13.8] \| \| 1.51 \| \| [0.38-6.03] \| \| \| 0.56 \| \| \| \| 36-48 months on PrEP \| 11.5 [8.0-16.5] \| 1.21 \| [0.71-1.62] \| 1.21 \| [0.72-2.04] \| 0.47 \| 15.1 [10.3-22.0] \| 1.26 \| [0.72-2.19] \| 0.42 \| 2.7 [0.7-10.8] \| \| 0.82 \| \| [0.14-4.68] \| \| \| 0.83 \| \| \| \| Pharyngeal gonorrhoea \|  \|  \|  \|  \|  \|  \|  \|  \|  \|  \|  \| \|  \| \|  \| \| \|  \| \| \| \| 0-12 months on PrEP \| 25.9 [21.1-31.7] \| Ref. \|  \| Ref. \|  \|  \| 29.1 [23.3-36.4] \| Ref. \|  \|  \| 16.9 [10.3-27.5] \| \| Ref. \| \|  \| \| \|  \| \| \| \| 12-24 months on PrEP \| 23.7 [19.0-29.5] \| 0.92 \| [0.67-1.25] \| 0.75 \| [0.55-1.03] \| 0.072 \| 26.4 [20.7-33.7] \| 0.78 \| [0.55-1.10] \| 0.16 \| 16.0 [9.5-27.1] \| \| 0.62 \| \| [0.29-1.31] \| \| \| 0.21 \| \| \| \| 24-36 months on PrEP \| 18.9 [14.7-24.4] \| 0.73 \| [0.52-1.03] \| 0.63 \| [0.45-0.88] \| **0.0072** \| 20.5 [15.3-27.3] \| 0.61 \| [0.42-0.89] \| **0.011** \| 14.9 [8.7-25.7] \| \| 0.74 \| \| [0.35-1.58] \| \| \| 0.44 \| \| \| \| 36-48 months on PrEP \| 20.1 [15.3-26.5] \| 0.78 \| [0.54-1.03] \| 0.80 \| [0.56-1.12] \| 0.20 \| 19.6 [14.0-27.3] \| 0.70 \| [0.46-1.04] \| 0.080 \| 21.5 [13.2-35.1] \| \| 1.29 \| \| [0.63-2.65] \| \| \| 0.48 \| \| \| \| **Infectious syphilis** \|  \|  \|  \|  \|  \|  \|  \|  \|  \|  \|  \| \|  \| \|  \| \| \|  \| \| \| \| 0-12 months on PrEP \| 11.7 [8.6-15.8] \| Ref. \|  \| Ref. \|  \|  \| 12.8 [9.2-18.0] \| Ref. \|  \|  \| 8.4 [4.2-16.9] \| \| Model did not converge. \| \| \| \| \| \| \| \| 12-24 months on PrEP \| 13.2 [9.8-17.7] \| 1.13 \| [0.72-1.77] \| 0.92 \| [0.59-1.42] \| 0.70 \| 11.8 [8.2-17.0] \| 0.79 \| [0.47-1.31] \| 0.36 \| 17.2 [10.3-28.5] \| \|  \| \|  \| \| \|  \| \| \| \| 24-36 months on PrEP \| 8.0 [5.4-11.9] \| 0.69 \| [0.40-1.15] \| 0.57 \| [0.34-0.95] \| **0.031** \| 8.0 [5.0-12.7] \| 0.54 \| [0.30-0.97] \| **0.038** \| 8.0 [3.8-16.9] \| \|  \| \|  \| \| \|  \| \| \| \| 36-48 months on PrEP \| 11.5 [8.0-16.5] \| 0.98 \| [0.59-1.15] \| 0.95 \| [0.59-1.53] \| 0.83 \| 15.1 [10.4-22.0] \| 1.15 \| [0.69-1.91] \| 0.59 \| 2.7 [0.7-10.8] \| \|  \| \|  \| \| \|  \| \| \| \| **Hepatitis C** \|  \|  \|  \|  \|  \|  \|  \|  \|  \|  \|  \| \|  \| \|  \| \| \|  \| \| \| \| 0-12 months on PrEP \| 1.8 [0.8-3.9] \| Ref. \|  \| Ref. \|  \|  \| 2.0 [0.8-4.8] \| * \|  \|  \| 1.2 [0.2-8.4] \| \| * \| \|  \| \| \|  \| \| \| \| 12-24 months on PrEP \| 2.5 [1.3-5.2] \| 1.42 \| [0.43-4.96] \| 0.54 \| [0.15-1.93] \| 0.34 \| 3.4 [1.7-6.9] \| 1.92 \| [0.62-5.98] \| 06 \| 0.0 [0.0-0.0] \| \| 0.00 \| \| [0.00-undef.] \| \| \| 1.00 \| \| \| \| 24-36 months on PrEP \| 0.7 [0.2-2.7] \| 0.38 \| [0.04-2.12] \| 0.14 \| [0.02-0.87] \| **0.035** \| 0.9 [0.2-3.7] \| 0.51 \| [0.10-2.67] \| 0 \| 0.0 [0.0-0.0] \| \| 0.00 \| \| [0.00-undef.] \| \| \| 1.00 \| \| \| \| 36-48 months on PrEP \| 0.4 [0.1-2.6] \| 0.23 \| [0.01-2.12] \| 0.08 \| [0.01-0.77] \| **0.029** \| 0.0 [0.0-0.0] \| 0.00 \| [0.00-.] \| 1.00 \| 1.4 [0.2-10.3] \| \| 1.11 \| \| [0.04-31.26] \| \| \| 0.95 \| \| \| |
| --- | --- | --- | --- | --- | --- | --- | --- | --- | --- | --- | --- | --- | --- | --- | --- | --- | --- | --- | --- | --- | --- | --- | --- | --- | --- | --- | --- | --- | --- | --- | --- | --- | --- | --- | --- | --- | --- | --- | --- | --- | --- | --- | --- | --- | --- | --- | --- | --- | --- | --- | --- | --- | --- | --- | --- | --- | --- | --- | --- | --- | --- | --- | --- | --- | --- | --- | --- | --- | --- | --- | --- | --- | --- | --- | --- | --- | --- | --- | --- | --- | --- | --- | --- | --- | --- | --- | --- | --- | --- | --- | --- | --- | --- | --- | --- | --- | --- | --- | --- | --- | --- | --- | --- | --- | --- | --- | --- | --- | --- | --- | --- | --- | --- | --- | --- | --- | --- | --- | --- | --- | --- | --- | --- | --- | --- | --- | --- | --- | --- | --- | --- | --- | --- | --- | --- | --- | --- | --- | --- | --- | --- | --- | --- | --- | --- | --- | --- | --- | --- | --- | --- | --- | --- | --- | --- | --- | --- | --- | --- | --- | --- | --- | --- | --- | --- | --- | --- | --- | --- | --- | --- | --- | --- | --- | --- | --- | --- | --- | --- | --- | --- | --- | --- | --- | --- | --- | --- | --- | --- | --- | --- | --- | --- | --- | --- | --- | --- | --- | --- | --- | --- | --- | --- | --- | --- | --- | --- | --- | --- | --- | --- | --- | --- | --- | --- | --- | --- | --- | --- | --- | --- | --- | --- | --- | --- | --- | --- | --- | --- | --- | --- | --- | --- | --- | --- | --- | --- | --- | --- | --- | --- | --- | --- | --- | --- | --- | --- | --- | --- | --- | --- | --- | --- | --- | --- | --- | --- | --- | --- | --- | --- | --- | --- | --- | --- | --- | --- | --- | --- | --- | --- | --- | --- | --- | --- | --- | --- | --- | --- | --- | --- | --- | --- | --- | --- | --- | --- | --- | --- | --- | --- | --- | --- | --- | --- | --- | --- | --- | --- | --- | --- | --- | --- | --- | --- | --- | --- | --- | --- | --- | --- | --- | --- | --- | --- | --- | --- | --- | --- | --- | --- | --- | --- | --- | --- | --- | --- | --- | --- | --- | --- | --- | --- | --- | --- | --- | --- | --- | --- | --- | --- | --- | --- | --- | --- | --- | --- | --- | --- | --- | --- | --- | --- | --- | --- | --- | --- | --- | --- | --- | --- | --- | --- | --- | --- | --- | --- | --- | --- | --- | --- | --- | --- | --- | --- | --- | --- | --- | --- | --- | --- | --- | --- | --- | --- | --- | --- | --- | --- | --- | --- | --- | --- | --- | --- | --- | --- | --- | --- | --- | --- | --- | --- | --- | --- | --- | --- | --- | --- | --- | --- | --- | --- | --- | --- | --- | --- | --- | --- | --- | --- | --- | --- | --- | --- | --- | --- | --- | --- | --- | --- | --- | --- | --- | --- | --- | --- | --- | --- | --- | --- | --- | --- | --- | --- | --- | --- | --- | --- | --- | --- | --- | --- | --- | --- | --- | --- | --- | --- | --- | --- | --- | --- | --- | --- | --- | --- | --- | --- | --- | --- | --- | --- | --- | --- | --- | --- | --- | --- | --- | --- | --- | --- | --- | --- | --- | --- | --- | --- | --- | --- | --- | --- | --- | --- | --- | --- | --- | --- | --- | --- | --- | --- | --- | --- | --- | --- | --- | --- | --- | --- | --- | --- | --- | --- | --- | --- | --- | --- | --- | --- | --- | --- | --- | --- | --- | --- | --- | --- | --- | --- | --- | --- | --- | --- | --- | --- | --- | --- | --- | --- | --- | --- | --- | --- | --- | --- | --- | --- | --- | --- | --- | --- | --- | --- | --- | --- | --- | --- | --- | --- | --- | --- | --- | --- | --- | --- | --- | --- | --- | --- | --- | --- | --- | --- | --- | --- | --- | --- | --- | --- | --- | --- | --- | --- | --- | --- | --- | --- | --- | --- | --- | --- | --- | --- | --- | --- | --- | --- | --- | --- | --- | --- | --- | --- | --- | --- | --- | --- | --- | --- | --- | --- | --- | --- | --- | --- | --- | --- | --- | --- | --- | --- | --- | --- | --- | --- | --- | --- | --- | --- | --- | --- | --- | --- | --- | --- | --- | --- | --- | --- | --- | --- | --- | --- | --- | --- | --- | --- | --- | --- | --- | --- | --- | --- | --- | --- | --- | --- | --- | --- | --- | --- | --- | --- | --- | --- | --- | --- | --- | --- | --- | --- | --- | --- | --- | --- | --- | --- | --- | --- | --- | --- | --- | --- | --- | --- | --- | --- | --- | --- | --- | --- | --- | --- | --- | --- | --- | --- | --- | --- | --- | --- | --- | --- | --- | --- | --- | --- | --- | --- | --- | --- | --- | --- | --- | --- | --- | --- | --- | --- | --- | --- | --- | --- | --- | --- | --- | --- | --- | --- | --- | --- | --- | --- | --- | --- | --- | --- | --- | --- | --- | --- | --- | --- | --- | --- | --- | --- | --- | --- | --- | --- | --- | --- | --- | --- | --- | --- | --- | --- | --- | --- | --- | --- | --- | --- | --- | --- | --- | --- | --- | --- | --- | --- | --- | --- | --- | --- | --- | --- | --- | --- | --- | --- | --- | --- | --- | --- | --- | --- | --- | --- | --- | --- | --- | --- | --- | --- | --- | --- | --- | --- | --- | --- | --- | --- | --- | --- | --- | --- | --- | --- | --- | --- | --- | --- | --- | --- | --- | --- | --- | --- | --- | --- | --- | --- | --- | --- | --- | --- | --- | --- | --- | --- | --- | --- | --- | --- | --- | --- | --- | --- | --- | --- | --- | --- | --- | --- | --- | --- | --- | --- | --- | --- | --- | --- | --- | --- | --- | --- | --- | --- | --- | --- | --- | --- | --- | --- | --- | --- | --- | --- | --- | --- | --- | --- | --- | --- | --- | --- | --- | --- | --- | --- | --- | --- | --- | --- | --- | --- | --- | --- | --- | --- | --- | --- | --- | --- | --- | --- | --- | --- | --- | --- | --- | --- | --- | --- | --- | --- | --- | --- | --- | --- | --- | --- | --- | --- | --- | --- | --- | --- | --- | --- | --- | --- | --- | --- | --- | --- | --- | --- | --- | --- | --- | --- | --- | --- | --- | --- | --- | --- | --- | --- | --- | --- | --- | --- | --- | --- | --- | --- | --- | --- | --- | --- | --- | --- | --- | --- | --- | --- | --- | --- | --- | --- | --- | --- | --- | --- | --- | --- | --- | --- | --- | --- | --- | --- | --- | --- | --- | --- | --- | --- | --- | --- | --- | --- | --- | --- | --- | --- | --- | --- | --- | --- | --- | --- | --- | --- | --- | --- | --- | --- | --- | --- | --- | --- | --- | --- | --- | --- | --- | --- | --- | --- | --- | --- | --- | --- | --- | --- | --- | --- | --- | --- | --- | --- | --- | --- | --- | --- | --- | --- | --- | --- | --- | --- | --- | --- | --- | --- | --- | --- | --- | --- | --- | --- | --- | --- | --- | --- | --- | --- | --- | --- | --- | --- | --- | --- | --- | --- | --- | --- | --- | --- | --- | --- | --- | --- | --- | --- | --- | --- | --- | --- | --- | --- | --- | --- | --- | --- | --- | --- | --- | --- | --- | --- | --- | --- | --- | --- | --- | --- | --- | --- | --- | --- | --- | --- | --- | --- | --- | --- | --- | --- | --- | --- | --- | --- | --- | --- | --- | --- | --- | --- | --- | --- | --- | --- | --- | --- | --- | --- | --- | --- | --- | --- | --- | --- | --- | --- | --- | --- | --- | --- | --- | --- | --- | --- | --- | --- | --- | --- | --- | --- | --- | --- | --- | --- | --- | --- | --- | --- | --- | --- | --- | --- | --- | --- | --- | --- | --- | --- | --- | --- | --- | --- | --- | --- | --- | --- | --- | --- | --- | --- | --- | --- | --- | --- | --- | --- | --- | --- | --- | --- | --- | --- | --- | --- | --- | --- | --- | --- | --- | --- | --- | --- | --- | --- | --- | --- | --- | --- | --- | --- | --- | --- | --- | --- | --- | --- | --- | --- | --- | --- | --- | --- | --- | --- | --- | --- | --- | --- | --- | --- | --- | --- | --- | --- | --- | --- | --- | --- | --- | --- | --- | --- | --- | --- | --- | --- | --- | --- | --- | --- | --- | --- | --- | --- | --- | --- | --- | --- | --- | --- | --- | --- | --- | --- | --- | --- | --- | --- | --- | --- | --- | --- | --- | --- | --- | --- | --- | --- | --- | --- | --- | --- | --- | --- | --- | --- | --- | --- | --- | --- | --- | --- | --- | --- | --- | --- | --- | --- | --- | --- | --- | --- | --- | --- | --- | --- | --- | --- | --- | --- | --- | --- | --- | --- | --- | --- | --- | --- | --- | --- | --- | --- | --- | --- | --- | --- | --- | --- | --- | --- | --- | --- | --- | --- | --- | --- | --- | --- | --- | --- | --- | --- | --- | --- | --- | --- | --- | --- | --- | --- | --- | --- | --- | --- | --- | --- | --- | --- | --- | --- | --- | --- | --- | --- | --- | --- | --- | --- | --- | --- | --- | --- | --- | --- | --- | --- | --- | --- | --- | --- | --- | --- | --- | --- | --- | --- | --- | --- | --- | --- | --- | --- | --- | --- | --- | --- | --- | --- | --- | --- | --- | --- | --- | --- | --- | --- | --- | --- | --- | --- | --- | --- | --- | --- | --- | --- | --- | --- | --- | --- | --- | --- | --- | --- | --- | --- | --- | --- | --- | --- | --- | --- | --- | --- | --- | --- | --- | --- | --- | --- | --- | --- | --- | --- | --- | --- | --- | --- | --- | --- | --- | --- | --- | --- | --- | --- | --- | --- | --- | --- | --- | --- | --- | --- | --- | --- | --- | --- | --- | --- | --- | --- | --- | --- | --- | --- | --- | --- | --- | --- | --- | --- | --- | --- | --- | --- | --- | --- | --- | --- | --- | --- | --- | --- | --- | --- | --- | --- | --- | --- | --- | --- | --- | --- | --- | --- | --- | --- | --- | --- | --- | --- | --- | --- | --- | --- | --- | --- | --- | --- | --- | --- | --- | --- | --- | --- | --- | --- | --- | --- | --- | --- | --- | --- | --- | --- | --- | --- | --- | --- | --- | --- | --- | --- | --- | --- | --- | --- | --- | --- | --- | --- | --- | --- | --- | --- | --- | --- | --- | --- | --- | --- | --- | --- | --- | --- | --- | --- | --- | --- | --- | --- | --- | --- | --- | --- | --- | --- | --- | --- | --- | --- | --- | --- | --- | --- | --- | --- | --- | --- | --- | --- | --- | --- | --- | --- | --- | --- | --- | --- | --- | --- | --- | --- | --- | --- | --- | --- | --- | --- | --- | --- | --- | --- | --- | --- | --- | --- | --- | --- | --- | --- | --- | --- | --- | --- | --- | --- | --- | --- | --- | --- | --- | --- | --- | --- | --- | --- | --- | --- | --- | --- | --- | --- | --- | --- | --- | --- | --- | --- | --- | --- | --- | --- | --- | --- | --- | --- | --- | --- | --- | --- | --- | --- | --- | --- | --- | --- | --- | --- | --- | --- | --- | --- | --- | --- | --- | --- | --- | --- | --- | --- | --- | --- | --- | --- | --- | --- | --- | --- | --- | --- | --- | --- | --- | --- | --- | --- | --- | --- | --- | --- | --- | --- | --- | --- | --- | --- | --- | --- | --- | --- | --- | --- | --- | --- | --- | --- | --- | --- | --- | --- | --- | --- | --- | --- | --- | --- | --- | --- | --- | --- | --- | --- | --- | --- | --- | --- | --- | --- | --- | --- | --- | --- | --- | --- | --- | --- | --- | --- | --- | --- | --- | --- | --- | --- | --- | --- | --- | --- | --- | --- | --- | --- | --- | --- | --- | --- | --- | --- | --- | --- | --- | --- | --- |
| Abbreviations: AMPrEP: Amsterdam PrEP demonstration project; CI: confidence interval; (a)IRR: (adjusted) incidence rate ratio; LGV: lymphogranuloma venereum; PrEP: pre-exposure prophylaxis; PY: person-years; Ref.: reference category; STI: sexually transmitted infection. ^a^Any chlamydia, gonorrhoea or infectious syphilis (stage 1, 2 or recent latent). ^b^Any anorectal chlamydia or gonorrhoea.  ^c^ Incidence rates were adjusted for age at baseline and time-updated yearly testing frequency for the respective STI (except for the aIRRs of LGV, which we corrected for the yearly testing frequency of anal chlamydia). Age and STI testing frequency were modelled as cubic splines with four knot for all STI except HCV. HCV was corrected for age as cubic splines with four knots and for the absolute number of HCV tests in a year (which ranged from 0 to 4).  ^d^p-value based on the Wald test  *We did not estimate IRRs for HCV among stratified daily and event-driven users due to the low number of incident infections that occurred. |
