# Supplementary material for: Sexual behaviour and incidence of sexually transmitted infections among men who have sex with men (MSM) using daily and event-driven pre-exposure prophylaxis (PrEP): Four-year follow-up of the Amsterdam PrEP (AMPrEP) demonstration project cohort
Source: PLoS Med. 2024 May 8;21(5):e1004328. doi: 10.1371/journal.pmed.1004328 (PMC11111007; doi:10.1371/journal.pmed.1004328)
Supplement: S7 Table — (DOCX) [file pmed.1004328.s007.docx]

| **S7 Table.** Incidence rate ratio for STIs per additional year on PrEP over four years of PrEP use, among 225 AMPrEP participants who never switched PrEP regimens, Amsterdam, the Netherlands, 2015-20 | | | | | | | | | | | | | | | | |
| --- | --- | --- | --- | --- | --- | --- | --- | --- | --- | --- | --- | --- | --- | --- | --- | --- |
|  | Total | | | | | |  | Daily PrEP | | | |  | Event-driven PrEP | | | |
|  | IR per 100 PY | Crude IRR  [95% CI] | | aIRR^c^ [95% CI] | | p-value^d^ |  | IR per 100 PY | aIRR^c^  [95% CI] | | p-value^d^ |  | IR per 100 PY | aIRR^c^  [95% CI] | | p-value^d^ |
| **Any STI**^a^ |  |  |  |  |  |  |  |  |  |  |  |  |  |  |  |  |
| 0-12 months on PrEP | 96.2 | Ref. |  | Ref. |  |  |  | 107.0 | Ref. |  |  |  | 55.0 | Ref. |  |  |
| 12-24 months on PrEP | 87.0 | 0.90 | [0.74-1.11] | 0.80 | [0.65-0.98] | **0.032** |  | 91.6 | 0.76 | [0.61-0.95] | **0.016** |  | 69.0 | 1.07 | [0.62-1.85] | 0.82 |
| 24-36 months on PrEP | 86.7 | 0.90 | [0.73-1.11] | 0.80 | [0.65-0.99] | **0.038** |  | 95.8 | 0.80 | [0.64-1.00] | **0.050** |  | 50.3 | 0.79 | [0.43-1.44] | 0.44 |
| 36-48 months on PrEP | 86.7 | 0.90 | [0.72-1.12] | 0.90 | [0.72-1.13] | 0.36 |  | 96.1 | 0.90 | [0.71-1.14] | 0.38 |  | 48.5 | 0.83 | [0.43-1.59] | 0.57 |
| **Any anal STI**^b^ |  |  |  |  |  |  |  |  |  |  |  |  |  |  |  |  |
| 0-12 months on PrEP | 66.1 | Ref. |  | Ref. |  |  |  | 75.3 | Ref. |  |  |  | 30.8 | Ref. |  |  |
| 12-24 months on PrEP | 60.3 | 0.91 | [0.71-1.17] | 0.82 | [0.64-1.05] | 0.12 |  | 66.8 | 0.80 | [0.62-1.04] | 0.092 |  | 34.5 | 1.02 | [0.48-2.17] | 0.96 |
| 24-36 months on PrEP | 65.6 | 0.99 | [0.77-1.27] | 0.89 | [0.70-1.15] | 0.38 |  | 72.7 | 0.87 | [0.67-1.13] | 0.29 |  | 37.0 | 1.12 | [0.52-2.40] | 0.77 |
| 36-48 months on PrEP | 61.8 | 0.94 | [0.72-1.22] | 0.91 | [0.70-1.18] | 0.48 |  | 69.9 | 0.90 | [0.68-1.19] | 0.47 |  | 29.1 | 0.83 | [0.35-1.95] | 0.67 |
| **Chlamydia** |  |  |  |  |  |  |  |  |  |  |  |  |  |  |  |  |
| Any chlamydia |  |  |  |  |  |  |  |  |  |  |  |  |  |  |  |  |
| 0-12 months on PrEP | 48.3 | Ref. |  | Ref. |  |  |  | 52.9 | Ref. |  |  |  | 30.8 | Ref. |  |  |
| 12-24 months on PrEP | 43.0 | 0.89 | [0.66-1.19] | 0.78 | [0.59-1.05] | 0.098 |  | 45.2 | 0.76 | [0.56-1.05] | 0.092 |  | 34.5 | 0.91 | [0.43-1.93] | 0.81 |
| 24-36 months on PrEP | 43.9 | 0.91 | [0.67-1.22] | 0.81 | [0.60-1.08] | 0.15 |  | 49.6 | 0.84 | [0.62-1.15] | 0.29 |  | 21.2 | 0.55 | [0.23-1.33] | 0.19 |
| 36-48 months on PrEP | 43.3 | 0.90 | [0.65-1.23] | 0.89 | [0.65-1.22] | 0.48 |  | 46.9 | 0.88 | [0.63-1.23] | 0.46 |  | 29.1 | 0.83 | [0.35-1.95] | 0.67 |
| Anal chlamydia |  |  |  |  |  |  |  |  |  |  |  |  |  |  |  |  |
| 0-12 months on PrEP | 39.2 | Ref. |  | Ref. |  |  |  | 43.7 | Ref. |  |  |  | 22.0 | Ref. |  |  |
| 12-24 months on PrEP | 34.1 | 0.87 | [0.62-1.21] | 0.78 | [0.56-1.08] | 0.13 |  | 36.5 | 0.75 | [0.53-1.07] | 0.11 |  | 24.6 | 0.94 | [0.39-2.28] | 0.89 |
| 24-36 months on PrEP | 36.0 | 0.92 | [0.66-1.28] | 0.83 | [0.60-1.15] | 0.26 |  | 41.0 | 0.85 | [0.60-1.20] | 0.36 |  | 15.9 | 0.62 | [0.22-1.74] | 0.37 |
| 36-48 months on PrEP | 38.9 | 0.99 | [0.70-1.39] | 1.00 | [0.71-1.40] | 0.99 |  | 43.7 | 1.00 | [0.70-1.43] | 1.0 |  | 19.4 | 0.80 | [0.29-2.25] | 0.67 |
| Urogenital chlamydia |  |  |  |  |  |  |  |  |  |  |  |  |  |  |  |  |
| 0-12 months on PrEP | 10.9 | Ref. |  | Ref. |  |  |  | 11.5 | Ref. |  |  |  | 8.8 | Ref. |  |  |
| 12-24 months on PrEP | 11.4 | 1.04 | [0.56-1.92] | 0.89 | [0.50-1.59] | 0.70 |  | 10.5 | 0.82 | [0.43-1.59] | 0.56 |  | 14.8 | 1.23 | [0.34-4.45] | 0.75 |
| 24-36 months on PrEP | 14.8 | 1.35 | [0.76-2.44] | 1.17 | [0.67-2.03] | 0.59 |  | 16.5 | 1.30 | [0.71-2.36] | 0.40 |  | 7.9 | 0.63 | [0.13-2.92] | 0.55 |
| 36-48 months on PrEP | 6.4 | 0.58 | [0.25-1.26] | 0.58 | [0.27-1.22] | 0.15 |  | 4.8 | 0.40 | [0.16-1.01] | 0.053 |  | 12.9 | 1.37 | [0.32-5.83] | 0.67 |

| Pharyngeal chlamydia |  |  |  |  |  |  |  |  |  |  |  |  |  |  |  |  |
| --- | --- | --- | --- | --- | --- | --- | --- | --- | --- | --- | --- | --- | --- | --- | --- | --- |
| 0-12 months on PrEP | 3.6 | Ref. |  | Ref. |  |  |  | 3.5 | Ref. |  |  |  | 4.4 | Model did not converge | | |
| 12-24 months on PrEP | 2.9 | 0.81 | [0.23-2.67] | 0.68 | [0.23-2.03] | 0.49 |  | 2.5 | 0.65 | [0.18-2.38] | 0.51 |  | 4.9 |  |  |  |
| 24-36 months on PrEP | 5.3 | 1.45 | [0.52-4.23] | 1.14 | [0.42-3.05] | 0.80 |  | 6.6 | 1.64 | [0.55-4.85] | 0.38 |  | 0.0 |  |  |  |
| 36-48 months on PrEP | 1.9 | 0.52 | [0.09-2.19] | 0.52 | [0.13-1.98] | 0.34 |  | 2.4 | 0.70 | [0.17-2.84] | 0.61 |  | 0.0 |  |  |  |
| LGV |  |  |  |  |  |  |  |  |  |  |  |  |  |  |  |  |
| 0-12 months on PrEP | 5.5 | Ref. |  | Ref. |  |  |  | 6.3 | Ref. |  |  |  | 2.2 | Model did not converge | | |
| 12-24 months on PrEP | 4.9 | 0.90 | [0.35-2.28] | 0.64 | [0.27-1.51] | 0.75 |  | 4.9 | 0.59 | [0.23-1.49] | 0.26 |  | 4.9 |  |  |  |
| 24-36 months on PrEP | 6.3 | 1.16 | [0.48-2.82] | 0.84 | [0.37-1.91] | 0.88 |  | 7.9 | 0.93 | [0.40-2.18] | 0.87 |  | 0.0 |  |  |  |
| 36-48 months on PrEP | 8.9 | 1.63 | [0.70-3.86] | 1.66 | [0.76-3.62] | 0.13 |  | 9.5 | 1.53 | [0.67-3.51] | 0.32 |  | 6.5 |  |  |  |
| **Gonorrhoea** |  |  |  |  |  |  |  |  |  |  |  |  |  |  |  |  |
| Any gonorrhoea |  |  |  |  |  |  |  |  |  |  |  |  |  |  |  |  |
| 0-12 months on PrEP | 55.2 | Ref. |  | Ref. |  |  |  | 62.7 | Ref. |  |  |  | 26.4 | Ref. |  |  |
| 12-24 months on PrEP | 46.5 | 0.84 | [0.64-1.11] | 0.74 | [0.57-0.98] | **0.035** |  | 51.3 | 0.72 | [0.54-0.96] | **0.027** |  | 27.1 | 1.03 | [0.44-2.39] | 0.95 |
| 24-36 months on PrEP | 49.7 | 0.90 | [0.68-1.19] | 0.80 | [0.61-1.06] | 0.12 |  | 55.5 | 0.78 | [0.58-1.05] | 0.096 |  | 26.5 | 1.05 | [0.44-2.51] | 0.91 |
| 36-48 months on PrEP | 48.5 | 0.88 | [0.65-1.18] | 0.89 | [0.66-1.19] | 0.43 |  | 54.0 | 0.87 | [0.64-1.20] | 0.40 |  | 25.9 | 0.94 | [0.38-2.37] | 0.90 |
| Anal gonorrhoea |  |  |  |  |  |  |  |  |  |  |  |  |  |  |  |  |
| 0-12 months on PrEP | 38.7 | Ref. |  | Ref. |  |  |  | 44.9 | Ref. |  |  |  | 45.5 | Ref. |  |  |
| 12-24 months on PrEP | 33.1 | 0.86 | [0.61-1.19] | 0.76 | [0.55-1.05] | 0.094 |  | 37.1 | 0.73 | [0.52-1.02] | 0.068 |  | 17.2 | 1.31 | [0.43-4.00] | 0.64 |
| 24-36 months on PrEP | 38.6 | 1.00 | [0.72-1.38] | 0.88 | [0.63-1.21] | 0.42 |  | 43.0 | 0.83 | [0.59-1.17] | 0.29 |  | 21.2 | 1.68 | [0.56-5.06] | 0.36 |
| 36-48 months on PrEP | 33.8 | 0.87 | [0.61-1.24] | 0.82 | [0.57-1.17] | 0.27 |  | 38.1 | 0.81 | [0.56-1.17] | 0.25 |  | 16.2 | 0.97 | [0.29-3.18] | 0.95 |
| Urogenital gonorrhoea |  |  |  |  |  |  |  |  |  |  |  |  |  |  |  |  |
| 0-12 months on PrEP | 9.1 | Ref. |  | Ref. |  |  |  | 10.4 | Ref. |  |  |  | 4.4 | Ref. |  |  |
| 12-24 months on PrEP | 7.4 | 0.81 | [0.39-1.67] | 0.75 | [0.38-1.47] | 0.41 |  | 7.4 | 0.66 | [0.32-1.39] | 0.28 |  | 7.4 | 1.63 | [0.26-10.11] | 0.60 |
| 24-36 months on PrEP | 7.9 | 0.87 | [0.41-1.79] | 0.79 | [0.40-1.56] | 0.49 |  | 9.9 | 0.86 | [0.43-1.75] | 0.69 |  | 0.0 | 0.00 | [0.00-undef.] | 1.00 |
| 36-48 months on PrEP | 12.1 | 1.33 | [0.67-2.62] | 1.46 | [0.75-2.81] | 0.26 |  | 14.3 | 1.56 | [0.78-3.11] | 0.21 |  | 3.2 | 0.78 | [0.07-9.33] | 0.84 |
| Pharyngeal gonorrhoea |  |  |  |  |  |  |  |  |  |  |  |  |  |  |  |  |
| 0-12 months on PrEP | 24.6 | Ref. |  | Ref. |  |  |  | 27.0 | Ref. |  |  |  | 15.4 | Ref. |  |  |
| 12-24 months on PrEP | 22.3 | 0.90 | [0.59-1.37] | 0.80 | [0.54-1.20] | 0.28 |  | 24.1 | 0.78 | [0.51-1.20] | 0.26 |  | 14.8 | 1.01 | [0.33-3.10] | 0.99 |
| 24-36 months on PrEP | 19.0 | 0.77 | [0.49-1.20] | 0.71 | [0.46-1.09] | 0.11 |  | 21.8 | 0.72 | [0.46-1.14] | 0.16 |  | 7.9 | 0.59 | [0.15-2.36] | 0.46 |
| 36-48 months on PrEP | 15.9 | 0.65 | [0.39-1.06] | 0.71 | [0.44-1.15] | 0.17 |  | 16.7 | 0.69 | [0.41-1.16] | 0.16 |  | 12.9 | 0.91 | [0.26-3.22] | 0.88 |

| **Infectious syphilis** |  |  |  |  |  |  |  |  |  |  |  |  |  |  |  |  |
| --- | --- | --- | --- | --- | --- | --- | --- | --- | --- | --- | --- | --- | --- | --- | --- | --- |
| 0-12 months on PrEP | 11.4 | Ref. |  | Ref. |  |  |  | 12.7 | Ref. |  |  |  | 6.5 | Ref. |  |  |
| 12-24 months on PrEP | 12.9 | 1.13 | [0.63-2.04] | 0.91 | [0.52-1.60] | 0.74 |  | 10.5 | 0.69 | [0.36-1.32] | 0.26 |  | 22.2 | 2.33 | [0.60-9.10] | 0.22 |
| 24-36 months on PrEP | 7.9 | 0.70 | [0.34-1.37] | 0.56 | [0.29-1.08] | 0.082 |  | 7.9 | 0.51 | [0.25-1.06] | 0.072 |  | 7.9 | 0.89 | [0.17-4.69] | 0.89 |
| 36-48 months on PrEP | 13.4 | 1.17 | [0.63-2.19] | 1.15 | [0.64-2.05] | 0.65 |  | 15.9 | 1.25 | [0.68-2.29] | 0.48 |  | 3.2 | 0.48 | [0.05-4.67] | 0.52 |
| **Hepatitis C** |  |  |  |  |  |  |  |  |  |  |  |  |  |  |  |  |
| 0-12 months on PrEP | 1.0 | Ref. |  | Ref. |  |  |  | * |  |  |  |  |  | * |  |  |
| 12-24 months on PrEP | 2.6 | 2.67 | [0.44-28.06] | 1.14 | [0.16-7.89] | 0.90 |  |  |  |  |  |  |  |  |  |  |
| 24-36 months on PrEP | 1.1 | 1.15 | [0.08-15.87] | 0.58 | [0.06-5.70] | 0.64 |  |  |  |  |  |  |  |  |  |  |
| 36-48 months on PrEP | 0.0 | 0.00 | [0.00-7.43] | 0.00 | [0.00-undef.] | 1.00 |  |  |  |  |  |  |  |  |  |  |
| Abbreviations: AMPrEP: Amsterdam PrEP demonstration project; CI: confidence interval; (a)IRR: (adjusted) incidence rate ratio; LGV: lymphogranuloma venereum;  PrEP: pre-exposure prophylaxis; PY: person-years; Ref.: reference category; STI: sexually transmitted infection. Undef.: undefined ^a^Any chlamydia, gonorrhoea or infectious syphilis (stage 1, 2 or recent latent). ^b^Any anorectal chlamydia or gonorrhoea.  ^c^ Incidence rates were adjusted for age at baseline and time-updated yearly testing frequency for the respective STI (except for the aIRRs of LGV, which we corrected for the yearly testing frequency of anal chlamydia). Age and STI testing frequency were modelled as cubic splines with four knot for all STI except HCV. HCV was corrected for age as cubic splines with four knots and for the absolute number of HCV tests in a year (which ranged from 0 to 4).  ^d^p-value based on the Wald test  *We did not estimate IRRs for HCV among stratified daily and event-driven users due to the low number of incident infections that occurred. | | | | | | | | | | | | | | | | |
