# Supplementary material for: Sexual behaviour and incidence of sexually transmitted infections among men who have sex with men (MSM) using daily and event-driven pre-exposure prophylaxis (PrEP): Four-year follow-up of the Amsterdam PrEP (AMPrEP) demonstration project cohort
Source: PLoS Med. 2024 May 8;21(5):e1004328. doi: 10.1371/journal.pmed.1004328 (PMC11111007; doi:10.1371/journal.pmed.1004328)
Supplement: S8 Table — (DOCX) [file pmed.1004328.s008.docx]

| **S8 Table.** PrEP regimen switch rates during up to four years of PrEP use, among 367 AMPrEP participants, Amsterdam, the Netherlands, 2015-20 | | | | | | | | | | | | | | | | | |
| --- | --- | --- | --- | --- | --- | --- | --- | --- | --- | --- | --- | --- | --- | --- | --- | --- | --- |
|  | **Any switch** | | | | |  | **Daily to event-driven** | | | | |  | **Event-driven to daily** | | | | |
|  | No. of PrEP users who switched ≥1 time | No. of switches | PY | Switch rate per 100PY [95% CI] | |  | No. of PrEP users who switched ≥1 time | No. of switches | PY | Switch rate per 100PY [95% CI] | |  | No. of PrEP users who switched ≥1 time | No. of switches | PY | Switch rate per 100PY [95% CI] | |
| Overall | 137 | 254 | 1248 | 20.4 | [18.0-23.0] |  | 109 | 141 | 906 | 15.5 | [13.2-18.4] |  | 94 | 113 | 342 | 33.0 | [27.5-39.7] |
| Year 1 | 74 | 92 | 352 | 26.1 | [21.3-32.1] |  | 45 | 47 | 258 | 17.8 | [13.7-24.2] |  | 44 | 45 | 94 | 47.7 | [35.6-63.9] |
| Year 2 | 53 | 64 | 329 | 19.4 | [15.2-24.8] |  | 34 | 34 | 243 | 14.0 | [10.0-19.6] |  | 30 | 30 | 87 | 34.6 | [24.2-49.5] |
| Year 3 | 43 | 54 | 310 | 17.4 | [13.3-22.7] |  | 31 | 31 | 224 | 13.8 | [9.7-19.7] |  | 22 | 23 | 86 | 26.7 | [17.8-40.2] |
| Year 4 | 34 | 44 | 256 | 17.2 | [12.8-23.1] |  | 28 | 29 | 181 | 16.0 | [11.1-23.1] |  | 14 | 15 | 75 | 19.9 | [12.0-33.0] |
| Abbreviations: AMPrEP: Amsterdam PrEP demonstration project; CI: confidence interval; PrEP: pre-exposure prophylaxis; PY: person years. | | | | | | | | | | | | | | | | | |
