# Supplementary material for: Sexual behaviour and incidence of sexually transmitted infections among men who have sex with men (MSM) using daily and event-driven pre-exposure prophylaxis (PrEP): Four-year follow-up of the Amsterdam PrEP (AMPrEP) demonstration project cohort
Source: PLoS Med. 2024 May 8;21(5):e1004328. doi: 10.1371/journal.pmed.1004328 (PMC11111007; doi:10.1371/journal.pmed.1004328)
Supplement: S9 Table — (DOCX) [file pmed.1004328.s009.docx]

| **S9 Table.** Factors associated with an earlier time until first stopping PrEP among 367 AMPrEP participants, Amsterdam, The Netherlands, 2015-20 | | | | | | |
| --- | --- | --- | --- | --- | --- | --- |
|  | Univariable^a^ | | |  | Multivariable^b^ | |
| Factors | HR | [95% CI] | p-value^c^ |  | aHR [95% CI] | p-value^c^ |
| **Sociodemographic characteristics** |  |  |  |  |  |  |
| Age (years) |  |  |  |  |  |  |
| ≥45 | ref. |  | <0.001 |  | ref. | **0.036** |
| 35-44 | 1.54 | [0.91-2.62] | |  | 0.73 [0.27-2.00] |  |
| <35 | 2.97 | [1.81-4.85] |  |  | 2.41 [1.06-4.57] |  |
| University/university of applied sciences degree | 0.60 | [0.39-0.92] | 0.019 |  | 0.44 [0.21-0.92] | **0.030** |
| Residency outside Amsterdam | 1.45 | [0.97-2.16] | 0.067 |  | 0.72 [0.34-1.54] | 0.40 |
| **Sexual behaviour** |  |  |  |  |  |  |
| Number of CAS acts with casual partners^d^ | |  |  |  |  |  |
| 0-2 | ref. |  | <0.001 |  | ref. | **0.039** |
| 3-9 | 0.46 | [0.27-0.79] |  |  | 0.49 [0.20-1.17] |  |
| 10-20 | 0.31 | [0.16-0.61] | |  | 0.08 [0.01-0.63] |  |
| ≥21 | 0.33 | [0.18-0.62] |  |  | 0.56 [0.22-1.40] |  |
| Any STI (past 3 months)^e^ | 0.58 | [0.30-1.11] | 0.10 |  | 0.48 [0.14-1.64] | 0.24 |
| **Mental health characteristics** | | |  |  |  |  |
| MHI-5 score <60 | 2.46 | [1.26-4.80] | 0.008 |  | 2.21 [1.05-4.63] | **0.036** |
| Abbreviations: (a)HR: (adjusted) hazard ratio; AMPrEP: Amsterdam PrEP demonstration project;  CAS: condomless anal sex; CI: confidence interval; MHI-5: five-item Mental Health Inventory;  PrEP: pre-exposure prophylaxis; STI: sexually transmitted infection. | | | | | | |
| ^a^Variables for univariable selection were selected a priori based on literature ^b^Variables included in multivariable analyses: age (years), education level (university/university of applied sciences degree or other), residence (in or outside Amsterdam), number of CAS acts with casual partners, any STI, MHI-5 score (<60 or ≥60)  ^c^Overall p-values were based on the likelihood-ratio test ^d^In the past 3 months, self-reported. Categorised into quartiles ^e^Any chlamydia, gonorrhoea or infectious syphilis (stage 1, 2 or recent latent) | | | | | | |
