# Supplementary material for: Sexual behaviour and incidence of sexually transmitted infections among men who have sex with men (MSM) using daily and event-driven pre-exposure prophylaxis (PrEP): Four-year follow-up of the Amsterdam PrEP (AMPrEP) demonstration project cohort
Source: PLoS Med. 2024 May 8;21(5):e1004328. doi: 10.1371/journal.pmed.1004328 (PMC11111007; doi:10.1371/journal.pmed.1004328)
Supplement: S1 Annex — (DOCX) [file pmed.1004328.s010.docx]

# **S1 Annex**: Acknowledgements of the H-TEAM consortium

H-TEAM members: T. van Benthem^^[[1]](#footnote-1)^^, D. Bons^^[[2]](#footnote-2)^^, G.J. de Bree^^[[3]](#footnote-3)^;^[[4]](#footnote-4)^^, P. Brokx^^[[5]](#footnote-5)^^, U. Davidovich^1;^[[6]](#footnote-6)^^, S. Hendriks^^[[7]](#footnote-7)^^, S.E. Geerlings^4^, M. Heidenrijk^3^, E. Hoornenborg^1^, J. Heijne^1;4^, P. Reiss^3;5^, A. van Sighem^^[[8]](#footnote-8)^^, M. van der Valk^4;8^, J. de Wit^^[[9]](#footnote-9)^^, P. Zantkuijl^7^.

H-TEAM Project Management: N. Schat^3^, L. Dol^3^.
H-TEAM additional collaborators: M. van Agtmael^^[[10]](#footnote-10)^^, J. Ananworanich^^[[11]](#footnote-11)^^, D. Van de Beek^^[[12]](#footnote-12)^^, G.E.L. van den Berk^^[[13]](#footnote-13)^^, D. Bezemer^8^, A. van Bijnen^7^, J.P. Bil^1^, W.L. Blok^12^, S. Bogers^4^, M. Bomers^10^, A. Boyd^1;8^, W. Brokking^^[[14]](#footnote-14)^^, D. Burger^^[[15]](#footnote-15)^^, K. Brinkman^13^, N. Brinkman^13^, M. de Bruin^^[[16]](#footnote-16)^^, S. Bruisten^1^, L. Coyer^1^, R. van Crevel^^[[17]](#footnote-17)^^, M. Dijkstra^1^, Y.T. van Duijnhoven^1^, A. van Eeden^14^, L. Elsenburg^14^, M.A.M. van den Elshout^1^, E. Ersan^^[[18]](#footnote-18)^^, P. E.V. Felipa^1^, T.B.H. Geijtenbeek^^[[19]](#footnote-19)^^, J. van Gool^1^, A. Goorhuis^4^, M. Groot^14^, C.A. Hankins^3^, A. Heijnen^^[[20]](#footnote-20)^;^[[21]](#footnote-21)^^, M.M.J Hillebregt^8^, M. Hommenga^1^, J.W. Hovius^4^, Y. Janssen^^[[22]](#footnote-22)^^, K. de Jong^1^, V. Jongen^1^, N.A. Kootstra^^[[23]](#footnote-23)^^, R.A. Koup^^[[24]](#footnote-24)^^, F.P. Kroon^^[[25]](#footnote-25)^^, T.J.W. van de Laar^^[[26]](#footnote-26)^;^[[27]](#footnote-27)^^, F. Lauw^^[[28]](#footnote-28)^^, M. M. van Leeuwen^5^, K. Lettinga^^[[29]](#footnote-29)^^, I. Linde^1^, D.S.E. Loomans^1^, I.M. van der Lubben^1^, J.T. van der Meer^4^, T. Mouhebati^7^, B.J. Mulder^1^, J. Mulder^^[[30]](#footnote-30)^^, F.J. Nellen^4^, A. Nijsters^7^, H. Nobel^4^, E.L.M. Op de Coul^^[[31]](#footnote-31)^^, E. Peters^10^, I.S. Peters^1^, T. van der Poll^4^, O. Ratmann^^[[32]](#footnote-32)^^, C. Rokx^^[[33]](#footnote-33)^^, M.F. Schim van der Loeff^1;^[[34]](#footnote-34)^^, W.E.M. Schoute^13^, J. Schouten^1^, J. Veenstra^29^, A. Verbon^33^, F. Verdult^5^, J. de Vocht^10^, H.J. de Vries^1;34;^[[35]](#footnote-35)^^, S. Vrouenraets^29^, M. van Vugt^4^, W.J. Wiersinga^4^, F.W. Wit^4;6^, L.R. Woittiez^4^, S. Zaheri^8^, P. Zantkuijl^7^, A. Żakowicz^^[[36]](#footnote-36)^^, M.C. van Zelm^^[[37]](#footnote-37)^^, H.M.L. Zimmermann^1^.

1. Department of Infectious Diseases, Public Health Service of Amsterdam, Amsterdam, the Netherlands [↑](#footnote-ref-1)
2. Trans United Europe, Amsterdam, The Netherlands [↑](#footnote-ref-2)
3. Department of Global Health, Amsterdam UMC – location AMC, and Amsterdam Institute for Global Health and Development, Amsterdam, the Netherlands [↑](#footnote-ref-3)
4. Department of Internal Medicine, Division of Infectious Diseases, Amsterdam UMC – location AMC, Amsterdam, the Netherlands [↑](#footnote-ref-4)
5. Dutch Association of PLHIV, Amsterdam, the Netherlands [↑](#footnote-ref-5)
6. Department of Social Psychology, University of Amsterdam, Amsterdam, the Netherlands [↑](#footnote-ref-6)
7. Soa Aids Nederland, Amsterdam, the Netherlands [↑](#footnote-ref-7)
8. Stichting HIV Monitoring, Amsterdam, the Netherlands [↑](#footnote-ref-8)
9. Department of Interdisciplinary Social Science: Public Health, Utrecht University, Utrecht, the Netherlands [↑](#footnote-ref-9)
10. Department of Internal Medicine, Amsterdam UMC – location VUMC, Amsterdam, the Netherlands [↑](#footnote-ref-10)
11. US Military HIV Research Program and the Henry M. Jackson Foundation for the Advancement of Military Medicine, Bethesda, United States [↑](#footnote-ref-11)
12. Center of Infection and Immunity Amsterdam (CINIMA), Department of Neurology, Amsterdam UMC – location AMC, Amsterdam, the Netherlands [↑](#footnote-ref-12)
13. Department of internal medicine, OLVG – location East, Amsterdam, the Netherlands [↑](#footnote-ref-13)
14. DC Klinieken, Amsterdam, the Netherlands [↑](#footnote-ref-14)
15. Department of Pharmacy, Radboud University Nijmegen Medical Center, Nijmegen, the Netherlands [↑](#footnote-ref-15)
16. Aberdeen Health Psychology Group, Institute of Applied Health Sciences, University of Aberdeen, Aberdeen, United Kingdom [↑](#footnote-ref-16)
17. Department of Internal Medicine, Radboud University Nijmegen Medical Center, Nijmegen, the Netherlands [↑](#footnote-ref-17)
18. Department of General Practice, Amsterdam UMC – location AMC, University of Amsterdam, Amsterdam, the Netherlands [↑](#footnote-ref-18)
19. Laboratory of Experimental Immunology, Amsterdam UMC – location AMC Amsterdam, the Netherlands [↑](#footnote-ref-19)
20. Sexology Center Amsterdam, Amsterdam, the Netherlands [↑](#footnote-ref-20)
21. GP practice Heijnen & de Meij, Amsterdam, the Netherlands [↑](#footnote-ref-21)
22. Primary Care Amsterdam and Almere (Elaa), Amsterdam, the Netherlands [↑](#footnote-ref-22)
23. Laboratory for Viral Immune Pathogenesis, Amsterdam UMC – location AMC Amsterdam, the Netherlands [↑](#footnote-ref-23)
24. Immunology Laboratory, Vaccine Research Center, National Institute of Allergy and Infectious Diseases, National Institutes of Health, Rockville, Maryland, USA [↑](#footnote-ref-24)
25. Department of Infectious Diseases, Leiden University Medical Center, Leiden, the Netherlands [↑](#footnote-ref-25)
26. Department of Medical Microbiology, OLVG, Amsterdam, the Netherlands [↑](#footnote-ref-26)
27. Department of Donor Medicine Research, Laboratory of Blood-borne Infections, Sanquin Research, Amsterdam, the Netherlands [↑](#footnote-ref-27)
28. Department of Internal Medicine, Medical Center Jan van Goyen, Amsterdam, the Netherlands [↑](#footnote-ref-28)
29. Department of Internal Medicine, OLVG – location West, Amsterdam, the Netherlands [↑](#footnote-ref-29)
30. Department of Internal Medicine, Slotervaart Hospital (former), Amsterdam, the Netherlands [↑](#footnote-ref-30)
31. Epidemiology and Surveillance Unit, Center for Infectious Disease Control, National Institute of Public Health and the Environment, the Netherlands [↑](#footnote-ref-31)
32. School of Public Health, Faculty of Medicine, Imperial College London, London, United Kingdom [↑](#footnote-ref-32)
33. Department of Internal Medicine and Infectious Diseases, Erasmus Medical Center, Rotterdam, the Netherlands [↑](#footnote-ref-33)
34. Center for Infection and Immunology, Amsterdam (CINIMA), Amsterdam UMC – location AMC, University of Amsterdam, Amsterdam, the Netherlands [↑](#footnote-ref-34)
35. Department of Dermatology, Amsterdam UMC – location AMC, University of Amsterdam, Amsterdam, the Netherlands [↑](#footnote-ref-35)
36. AIDS Healthcare Foundation, Amsterdam, the Netherlands [↑](#footnote-ref-36)
37. Department of Virology, Erasmus Medical Center, Rotterdam, the Netherlands [↑](#footnote-ref-37)
