# Supplementary material for: Sexual behaviour and incidence of sexually transmitted infections among men who have sex with men (MSM) using daily and event-driven pre-exposure prophylaxis (PrEP): Four-year follow-up of the Amsterdam PrEP (AMPrEP) demonstration project cohort
Source: PLoS Med. 2024 May 8;21(5):e1004328. doi: 10.1371/journal.pmed.1004328 (PMC11111007; doi:10.1371/journal.pmed.1004328)
Supplement: S1 STROBE checklist — (DOCX) [file pmed.1004328.s011.docx]

S1 STROBE Statement—checklist of items that should be included in reports of observational studies

|  | | | | Item No. | | Recommendation | Section | Relevant text from manuscript | | |  |
| --- | --- | --- | --- | --- | --- | --- | --- | --- | --- | --- | --- |
| **Title and abstract** | | | | 1 | | (*a*) Indicate the study’s design with a commonly used term in the title or the abstract | Abstract, paragraph 1 | demonstration project cohort | | |  |
|  |  |  |  |  |  | (*b*) Provide in the abstract an informative and balanced summary of what was done and what was found | Abstract, paragraph 2 and 3 | The Amsterdam PrEP demonstration project (AMPrEP) was a prospective, closed cohort study, providing oral daily PrEP and event-driven PrEP to MSM and transgender women in 2015-2020. Participants could choose their PrEP regimen, and could switch at each three-monthly visit. STI testing occurred at and, upon request, in-between 3-monthly study visits. We assessed changes in numbers of sex partners and condomless anal sex (CAS) acts with casual partners over time using negative binomial regression, adjusted for age. We assessed HIV incidence and changes in incidence rates (IR) of any STI (i.e., chlamydia, gonorrhoea, or infectious syphilis), and individual STIs over time using Poisson regression, adjusted for age and testing frequency.  367 participants (365 MSM) commenced PrEP and were followed for a median 3.9 years (interquartile range [IQR]=3.4-4.0). Median age was 40 years (IQR=32-48), 315 participants (85.8%) self-declared ethnicity as white and 280 (76.3%) had a university or university of applied sciences degree. Overall median number of sex partners (past 3 months) was 13 (IQR=6-26) and decreased per additional year on PrEP (adjusted rate ratio [aRR]=0.86/year, 95% confidence interval [CI]=0.83-0.88). Overall median number of CAS acts with casual partners (past 3 months) was 10 (IQR=3-20.5) and also decreased (aRR=0.92/year, 95%CI=0.88-0.97). We diagnosed any STI in 1092 consultations during 1258 person years, resulting in an IR of 87/100 person years (95%CI=82-92). IRs of any STI did not increase over time for daily PrEP or event-driven PrEP users. Two daily PrEP users, and no event-driven PrEP users, were diagnosed with HIV during their first year on PrEP. Study limitations include censoring follow-up due to COVID-19 measures and an underrepresentation of younger, non-white, practically educated and transgender individuals. | | |  |
| Introduction | | | | | | | |  | | |  |
| Background/rationale | | | | 2 | | Explain the scientific background and rationale for the investigation being reported | Introduction, paragraph 2 | To the best of our knowledge, there are no studies with longer-follow-up time evaluating behavioural trends and STI incidence rates among MSM on PrEP. However, since many PrEP users are expected to use PrEP for several years, such information would be needed to inform policy makers and clinicians of current and future PrEP programmes. | | |  |
| Objectives | | | | 3 | | State specific objectives, including any prespecified hypotheses | Introduction, paragraph 2 | Therefore, we prospectively assessed sexual behaviour and incidence rates of HIV and other STIs, including hepatitis C virus (HCV), among MSM on PrEP for up to four years. We also assessed switching between the daily and event-driven regimen, PrEP discontinuation and adherence to PrEP. | | |  |
| Methods | | | | | | | |  | | |  |
| Study design | | | | 4 | | Present key elements of study design early in the paper | Methods, Study design | The Amsterdam PrEP demonstration project (AMPrEP) was an open-label demonstration study conducted between 3 August 2015 and 1 December 2020 that included MSM and transgender women. Participants were offered a free-of-charge oral coformulation of emtricitabine and tenofovir disoproxil 200/245 mg (TDF/FTC) to be used as daily or event-driven PrEP. | | |  |
| Setting | | | | 5 | | Describe the setting, locations, and relevant dates, including periods of recruitment, exposure, follow-up, and data collection | Methods, Study design | The Amsterdam PrEP demonstration project (AMPrEP) was an open-label demonstration study conducted between 3 August 2015 and 1 December 2020 that included MSM and transgender women. Participants were offered a free-of-charge oral coformulation of emtricitabine and tenofovir disoproxil 200/245 mg (TDF/FTC) to be used as daily or event-driven PrEP. The study design, aim and procedures have been described previously, and analyses of the first 24 months of follow-up and HCV incidence have been published before. Briefly, participants attended 3-monthly study visits at the Centre for Sexual Health of the Public Health Service of Amsterdam, the Netherlands. Eligible were MSM and transgender women without HIV, who were ≥18 years old and had, in the 6 months prior to screening, a substantial likelihood to acquire HIV sexually. Switching between daily PrEP and event-driven PrEP was allowed at each 3-monthly study visit. All AMPrEP participants provided samples for HIV, HCV and STI testing at each study visit. We requested participants to also provide blood for dried blood spots (DBS) to measure adherence at the 3 or 6 and 12, 24 and 48 months study visits. We tested for HCV every 12 months until December 2016, and every 6 months thereafter. | | |  |
| Participants | | | | 6 | | (*a*) *Cohort study*—Give the eligibility criteria, and the sources and methods of selection of participants. Describe methods of follow-up | Methods, Study design | Eligible were MSM and transgender women without HIV, who were ≥18 years old and had, in the 6 months prior to screening, a substantial likelihood to acquire HIV sexually. | | |  |
|  |  |  |  |  |  | (*b*) *Cohort study*—For matched studies, give matching criteria and number of exposed and unexposed | n/a |  | | |  |
| Variables | | | | 7 | | Clearly define all outcomes, exposures, predictors, potential confounders, and effect modifiers. Give diagnostic criteria, if applicable | Methods, Measures; Methods, Outcomes; | Sociodemographic, psychosocial, clinical and behavioural characteristics were collected via questionnaires. Sociodemographics collected at inclusion in AMPrEP were age, gender identity, self-declared ethnicity, place of residency, education level, employment status, income level, living situation, relationship status and sexual preference. Behavioural and clinical characteristics included history of condomless anal sex and bacterial STIs in the 6 months prior to inclusion. Self-reported number of sex partners and anal sex acts, including partner type and condom use, were recorded three-monthly. Participants self-reported half-yearly whether they engaged in chemsex, defined as the use of γ-hydroxybutyrate/γ-butyrolactone, methamphetamine or mephedrone prior to or during sex.  Psychosocial determinants were measured yearly. Sexual compulsivity was measured using the sexual compulsivity scale, with a score ≥24 being indicative of a greater impact of sexual thoughts on daily functioning and of an inability to control sexual thoughts or behaviours. Sexual satisfaction was measured using the New Sexual Satisfaction Scale (NSSS) on a scale from 20-100. Symptoms of depression or anxiety were assessed using the Mental Health Inventory-5 (MHI-5) score, where a score of <60 indicated symptoms of depression or anxiety. The Alcohol Use Disorders Identification Test (AUDIT) and Drug Use Disorder Identification Test (DUDIT) questionnaires were used to assess problematic alcohol and drug use, respectively; scores ≥8 are interpreted as indicative of alcohol-related or drug-related problems.  We assessed the number of sex partners, number of anal sex acts, and number of condomless anal sex (CAS) acts with casual partners in the past 3 months at each study visit.  We assessed the number of diagnoses of chlamydia, gonorrhoea, infectious syphilis (stage 1, 2 and recent latent syphilis), HCV and HIV. We calculated incidence rates (IRs) as the number of visits with a diagnosis (including repeat infections) divided by the person years (PY) of follow-up. Diagnoses were laboratory-confirmed infections from samples taken during study visits or additional visits at the Centre for Sexual Health during follow-up. We defined any STI as having one or more bacterial STIs (i.e., chlamydia, gonorrhoea or infectious syphilis) at a visit. We stratified chlamydia and gonorrhoea infections by anatomical site (i.e., anal, urogenital, or pharyngeal), and defined any anal STI as having anal chlamydia or anal gonorrhoea. In calculating PY for IRs of bacterial STI, we assumed that infection occurred at the date of positive test and follow-up time recontinued after infection. We defined incident HCV infections according to clinical practice guidelines, and distinguished between primary infections and reinfections. In calculating PY for IRs of HCV, we assumed that the infection occurred midway between the last negative and first positive test. Follow-up time stopped after infection and continued after confirmed sustained virologic response. In calculating PY for IRs of HIV, we assumed that infection occurred midway between the last negative and first positive test, and follow-up time stopped after infection.  We evaluated the number and rates of any switch between regimens as well as switch from daily PrEP to event-driven PrEP and vice versa. We also evaluated the number and rate of PrEP discontinuations, which were defined as one of the following: (a) a duration between study visits lasting at least nine months without self-reporting continuing PrEP elsewhere during this period, (b) reporting not having taken PrEP for at least 3 months (regardless of visit attendance), (c) attending a formal study exit visit without self-reporting continuation of PrEP elsewhere, or (d) being lost-to-follow up. Loss-to-follow-up was defined as not attending a study visit in the nine months prior to 15 March 2020 (i.e., the start of the COVID-19 lockdown measures in the Netherlands), whilst not having completed the 48-month visit. Finally, we calculated the proportion of participants who still used PrEP at 48 months after enrolment among those who could have reached 48 months of follow-up before censoring.  We calculated median levels of intracellular tenofovir diphosphate (TFV-DP) in dried blood spots and corresponding IQRs among daily PrEP users and report the proportion of daily PrEP users with good adherence (TFV-DP ≥700 fmol/punch). We do not report these outcomes for event-driven PrEP users, since TFV-DP does not indicate prevention-effective adherence to event-driven PrEP. | | |  |
| Data sources/ measurement | | | | 8* | | For each variable of interest, give sources of data and details of methods of assessment (measurement). Describe comparability of assessment methods if there is more than one group | Methods, Measures;  Methods, Outcomes, paragraph 2;  Methods, Laboratory methods | Sociodemographic, psychosocial, clinical and behavioural characteristics were collected via questionnaires. Sociodemographics collected at inclusion in AMPrEP were age, gender identity, self-declared ethnicity, place of residency, education level, employment status, income level, living situation, relationship status and sexual preference. Behavioural and clinical characteristics included history of condomless anal sex and bacterial STIs in the 6 months prior to inclusion. Self-reported number of sex partners and anal sex acts, including partner type and condom use, were recorded three-monthly. Participants self-reported half-yearly whether they engaged in chemsex, defined as the use of γ-hydroxybutyrate/γ-butyrolactone, methamphetamine or mephedrone prior to or during sex.  Psychosocial determinants were measured yearly. Sexual compulsivity was measured using the sexual compulsivity scale, with a score ≥24 being indicative of a greater impact of sexual thoughts on daily functioning and of an inability to control sexual thoughts or behaviours. Sexual satisfaction was measured using the New Sexual Satisfaction Scale (NSSS) on a scale from 20-100. Symptoms of depression or anxiety were assessed using the Mental Health Inventory-5 (MHI-5) score, where a score of <60 indicated symptoms of depression or anxiety. The Alcohol Use Disorders Identification Test (AUDIT) and Drug Use Disorder Identification Test (DUDIT) questionnaires were used to assess problematic alcohol and drug use, respectively; scores ≥8 are interpreted as indicative of alcohol-related or drug-related problems.  […]  We assessed the number of diagnoses of chlamydia, gonorrhoea, infectious syphilis (stage 1, 2 and recent latent syphilis), HCV and HIV. […] Diagnoses were laboratory-confirmed infections from samples taken during study visits or additional visits at the Centre for Sexual Health during follow-up.  […]  Laboratory methods were described previously. For DBS analyses of intracellular TFV-DP, the 48 month samples were measured using a 50:50 methanol:water extraction. Results were divided by 1.138 in order to compare them with the previous 70:30 extractions. | | |  |
| Bias | | | | 9 | | Describe any efforts to address potential sources of bias | Methods, Outcomes, paragraph 2;  Methods, Outcomes, paragraph 3;  Methods, Outcomes, paragraph 4;  Methods, Statistical methods, paragraph 2;  Methods, Statistical methods, paragraph 3;  Methods, Statistical methods, paragraph 5 | Diagnoses were laboratory-confirmed infections from samples taken during study visits or additional visits at the Centre for Sexual Health during follow-up.  […]  Loss-to-follow-up was defined as not attending a study visit in the nine months prior to 15 March 2020 (i.e., the start of the COVID-19 lockdown measures in the Netherlands), whilst not having completed the 48-month visit. Finally, we calculated the proportion of participants who still used PrEP at 48 months after enrolment among those who could have reached 48 months of follow-up before censoring.  […]  We calculated median levels of intracellular tenofovir diphosphate (TFV-DP) in dried blood spots and corresponding IQRs among daily PrEP users and report the proportion of daily PrEP users with good adherence (TFV-DP ≥700 fmol/punch). We do not report these outcomes for event-driven PrEP users, since TFV-DP does not indicate prevention-effective adherence to event-driven PrEP.  […]  We provide unadjusted estimates and estimates adjusted for age at baseline. We initially modelled age as a categorical variable based on its non-linear association with sexual behaviour outcomes. Following peer-review, we modelled age as restricted cubic splines with four knots at the 5th, 35th, 65th and 95th percentiles, to minimise loss of information.  […]  We provide unadjusted estimates and estimates adjusted for age at baseline and individual yearly STI testing frequency (time-updated). We initially modelled age and STI testing frequency as categorical variables based on their non-linear association with any STI incidence. Following peer-review, we modelled both variables as restricted cubic splines with four knots at the 5th, 35th, 65th and 95th percentiles, to minimise loss of information.  […]  In sensitivity analyses, we re-ran the models on STI incidence and sexual behaviour using follow-up time including periods between PrEP discontinuation and re-initiation as periods with missing data. Additionally, we re-ran the models on STI incidence and sexual behaviour among participants who never switched PrEP regimens. | | |  |
| Study size | | | | 10 | | Explain how the study size was arrived at | Methods, Study design;  Results, Participant characteristics and duration of follow-up | The study design, aim and procedures have been described previously.  Between August 3, 2015 and May 31, 2016, 376 participants were enrolled. Of these, nine (2.4%) were excluded from analyses because they did not attend any follow-up visits.  […]  Of 282 participants who could have reached 48 months of follow-up before censoring, 192 (68%) still used PrEP after 48 months. | | |  |
| Quantitative variables | | 11 | | | Explain how quantitative variables were handled in the analyses. If applicable, describe which groupings were chosen and why | | Methods, Outcomes;  Methods, Laboratory methods | | Outcomes  We assessed the number of sex partners, number of anal sex acts, and number of condomless anal sex (CAS) acts with casual partners in the past 3 months at each study visit.  We assessed the number of diagnoses of chlamydia, gonorrhoea, infectious syphilis (stage 1, 2 and recent latent syphilis), HCV and HIV. We calculated incidence rates (IRs) as the number of visits with a diagnosis (including repeat infections) divided by the person years (PY) of follow-up. Diagnoses were laboratory-confirmed infections from samples taken during study visits or additional visits at the Centre for Sexual Health during follow-up. We defined any STI as having one or more bacterial STIs (i.e., chlamydia, gonorrhoea or infectious syphilis) at a visit. We stratified chlamydia and gonorrhoea infections by anatomical site (i.e., anal, urogenital, or pharyngeal), and defined any anal STI as having anal chlamydia or anal gonorrhoea. In calculating PY for IRs of bacterial STI, we assumed that infection occurred at the date of positive test and follow-up time recontinued after infection. We defined incident HCV infections according to clinical practice guidelines, and distinguished between primary infections and reinfections. In calculating PY for IRs of HCV, we assumed that the infection occurred midway between the last negative and first positive test. Follow-up time stopped after infection and continued after confirmed sustained virologic response. In calculating PY for IRs of HIV, we assumed that infection occurred midway between the last negative and first positive test, and follow-up time stopped after infection.  We evaluated the number and rates of any switch between regimens as well as switch from daily PrEP to event-driven PrEP and vice versa. We also evaluated the number and rate of PrEP discontinuations, which were defined as one of the following: (a) a duration between study visits lasting at least nine months without self-reporting continuing PrEP elsewhere during this period, (b) reporting not having taken PrEP for at least 3 months (regardless of visit attendance), (c) attending a formal study exit visit without self-reporting continuation of PrEP elsewhere, or (d) being lost-to-follow up. Loss-to-follow-up was defined as not attending a study visit in the nine months prior to 15 March 2020 (i.e., the start of the COVID-19 lockdown measures in the Netherlands), whilst not having completed the 48-month visit. Finally, we calculated the proportion of participants who still used PrEP at 48 months after enrolment among those who could have reached 48 months of follow-up before censoring.  We calculated median levels of intracellular tenofovir diphosphate (TFV-DP) in dried blood spots and corresponding IQRs among daily PrEP users and report the proportion of daily PrEP users with good adherence (TFV-DP ≥700 fmol/punch). We do not report these outcomes for event-driven PrEP users, since TFV-DP does not indicate prevention-effective adherence to event-driven PrEP.  […]  Laboratory methods were described previously. For DBS analyses of intracellular TFV-DP, the 48 month samples were measured using a 50:50 methanol:water extraction. Results were divided by 1.138 in order to compare them with the previous 70:30 extractions. | | |
| Statistical methods | | 12 | | | (*a*) Describe all statistical methods, including those used to control for confounding | | Methods, Statistical methods | | We excluded participants without any follow-up study visits. Follow-up began at PrEP initiation (i.e., ‘baseline’) and continued until 48-months of individual follow-up, last study visit, HIV diagnosis, or 15 March 2020, whichever occurred first. We excluded periods between PrEP discontinuation (as described above) and PrEP re-initiation from follow-up time. We presented analyses for both the overall study population and stratified on daily PrEP or event-driven PrEP. PrEP regimen was included as a time-updated variable.  To analyse changes in sexual behaviour, we report the median and interquartile ranges (IQR) of sexual behaviour outcomes for each study visit. We modelled the year-on-year change in mean sexual behaviour outcomes (excluding baseline visits) using a mixed-effects negative binomial regression model with a random intercept and random slope on the participant-level to account for between-individual variability at baseline and during follow-up, respectively, and robust standard errors to ensure variance corresponded to individuals. We report relative ratios (RR) and corresponding 95% confidence intervals (CI), and used a Wald χ2 test to test for changes over time. We provide unadjusted estimates and estimates adjusted for age at baseline. We initially modelled age as a categorical variable based on its non-linear association with sexual behaviour outcomes. Following peer-review, we modelled age as restricted cubic splines with four knots at the 5th, 35th, 65th and 95th percentiles, to minimise loss of information.  We calculated STI IRs per 100PY of follow-up and corresponding 95%CI based on a Poisson distribution. To obtain insight into regimen choice, regimen switching and STI incidence, we calculated the IR difference between daily PrEP and event-driven PrEP users among all participants and, after peer-review, additionally for the subset of participants who ever switched PrEP regimens. We report two-sided p-values for the IR difference. To analyse changes in bacterial STI incidence, we calculated STI IRs per 100PY for each 3-monthly follow-up period. We examined whether there was a linear association between time on PrEP and STI incidence using Poisson regression, and thereafter whether STI incidence varied between three-monthly periods within each year of PrEP use, jointly. Because there was a non-linear association between time on PrEP and STI incidence, but no statistical evidence for variation in STI incidence within years (p=0.22), we modelled the change in STI IRs in years 2, 3 and 4 compared to the first year on PrEP. We used Poisson regression with a gamma-distributed frailty, and added a random intercept on the individual level. We report incidence rate ratios (IRR) and corresponding 95%CI per year, and used a Wald χ2 test to test for changes compared to the first year on PrEP. We provide unadjusted estimates and estimates adjusted for age at baseline and individual yearly STI testing frequency (time-updated). We initially modelled age and STI testing frequency as categorical variables based on their non-linear association with any STI incidence. Following peer-review, we modelled both variables as restricted cubic splines with four knots at the 5th, 35th, 65th and 95th percentiles, to minimise loss of information. We did not model change in HIV incidence rates over time owing to the low number of infections.  To analyse changes in PrEP use, we calculated regimen switch rates per 100PY and 95%CI based on a Poisson distribution, and calculated linear change in switch rates per year as switch rate ratio. We calculated the total number of PrEP discontinuations (including discontinuations after re-initiating) and median time until first discontinuing PrEP. We evaluated factors associated with time until first stopping PrEP using multivariable Cox regression. The factors were selected a priori and related to: sociodemographics (age, education level and place of residence), sexual behaviour and STI (number of CAS acts with casual partners, any bacterial STI in the past 3 months), and mental wellbeing. We included education level and place of residency as time-fixed variables and all other variables as time-updated. Data from (half-)yearly questionnaires were carried backwards for study visits that occurred in the past 6 and 12 months, respectively.  In sensitivity analyses, we re-ran the models on STI incidence and sexual behaviour using follow-up time including periods between PrEP discontinuation and re-initiation as periods with missing data. Additionally, we re-ran the models on STI incidence and sexual behaviour among participants who never switched PrEP regimens.  We defined significance at a p-value <0.05. All statistical analyses were performed in STATA version 17.0 (StataCorp, College Station, Texas, USA). | | |
|  |  |  |  |  | (*b*) Describe any methods used to examine subgroups and interactions | | Methods, Statistical methods, paragraph 1;  Methods, Statistical methods, paragraph 3 | | We presented analysis for both the overall study population and stratified on daily PrEP or event-driven PrEP. PrEP regimen was included as a time-updated variable.  […]  To obtain insight into regimen choice, regimen switching and STI incidence, we calculated the IR difference between daily PrEP and event-driven PrEP users among all participants and, after peer-review, additionally for the subset of participants who ever switched PrEP regimens. We report two-sided p-values for the IR difference. | | |
|  |  |  |  |  | (*c*) Explain how missing data were addressed | | Methods, Statistical methods, paragraph 4;  Methods, Statistical methods, paragraph 5 | | Data from (half-)yearly questionnaires were carried backwards for study visits that occurred in the past 6 and 12 months, respectively.  […]  In sensitivity analyses, we re-ran the models on STI incidence and sexual behaviour using follow-up time including periods between PrEP discontinuation and re-initiation as periods with missing data | | |
|  |  |  |  |  | (*d*) *Cohort study*—If applicable, explain how loss to follow-up was addressed | | Methods, Outcomes, paragraph 3;  Methods, Statistical methods, paragraph 1 | | Loss-to-follow-up was defined as not attending a study visit in the nine months prior to 15 March 2020 (i.e., the start of the COVID-19 lockdown measures in the Netherlands), whilst not having completed the 48-month visit. Finally, we calculated the proportion of participants who still used PrEP at 48 months after enrolment among those who could have reached 48 months of follow-up before censoring.  […]  We excluded participants without any follow-up study visits. Follow-up began at PrEP initiation (i.e., ‘baseline’) and continued until 48-months of individual follow-up, last study visit, HIV diagnosis, or 15 March 2020, whichever occurred first. We excluded periods between PrEP discontinuation (as described above) and PrEP re-initiation from follow-up time. | | |
|  |  |  |  |  | (*e*) Describe any sensitivity analyses | | Methods, Statistical methods, paragraph 5 | | In sensitivity analyses, we re-ran the models on STI incidence and sexual behaviour using follow-up time including periods between PrEP discontinuation and re-initiation as periods with missing data. | | |
| Results | | | | | | | | | | | |
| Participants | | 13* | | | (a) Report numbers of individuals at each stage of study—eg numbers potentially eligible, examined for eligibility, confirmed eligible, included in the study, completing follow-up, and analysed | | Methods, Study design;  Results; Participant characteristics and duration of follow-up | | The study design, aim and procedures have been described previously.  […]  Between August 3, 2015 and May 31, 2016, 376 participants were enrolled. Of these, nine (2.4%) were excluded from analyses because they did not attend any follow-up visits. Of the 367 included participants, 365 were MSM and two identified as transgender women. Median age at baseline was 40 years (IQR=32-48), 315/367 (85.8%) self-declared to be white and 280/367 (76.3%) had a university/university of applied sciences degree (Table 1). The median follow-up time was 3.9 years (IQR=3.4-4.0), totalling 1258PY of observation. Of 282 participants who could have reached 48 months of follow-up before censoring, 192 (68%) still used PrEP after 48 months. | | |
|  |  |  |  |  | (b) Give reasons for non-participation at each stage | | Methods, Study design;  Results; Participant characteristics and duration of follow-up  Results; Discontinuation of PrEP use | | The study design, aim and procedures have been described previously.  […]  Between August 3, 2015 and May 31, 2016, 376 participants were enrolled. Of these, nine (2.4%) were excluded from analyses because they did not attend any follow-up visits. Of the 367 included participants, 365 were MSM and two identified as transgender women. Median age at baseline was 40 years (IQR=32-48), 315/367 (85.8%) self-declared to be white and 280/367 (76.3%) had a university/university of applied sciences degree (Table 1). The median follow-up time was 3.9 years (IQR=3.4-4.0), totalling 1258PY of observation. Of 282 participants who could have reached 48 months of follow-up before censoring, 192 (68%) still used PrEP after 48 months.  […]  We observed 112 PrEP stops among 98/367 (27%) participants; 31 stops were subsequently followed by a restart. 43 participants had a formal study exit visit and another 43 were lost-to-follow-up. We registered 17 gaps of more than 9 months between study visits, and nine participants reported not having used PrEP for a period of at least three months, despite continuing study participation. | | |
|  |  |  |  |  | (c) Consider use of a flow diagram | | Methods, Study design; | | The study design, aim and procedures have been described previously. | | |
| Descriptive data | | 14* | | | (a) Give characteristics of study participants (eg demographic, clinical, social) and information on exposures and potential confounders | | Results, Participant characteristics and duration of follow-up | | Of the 367 included participants, 365 were MSM and two identified as transgender women. Median age at baseline was 40 years (IQR=32-48), 315/367 (85.8%) self-declared to be white and 280/367 (76.3%) had a university/university of applied sciences degree (Table 1). | | |
|  |  |  |  |  | (b) Indicate number of participants with missing data for each variable of interest | | Table 1 | |  | | |
|  |  |  |  |  | (c) *Cohort study*—Summarise follow-up time (eg, average and total amount) | | Results, Participant characteristics and duration of follow-up | | The median follow-up time was 3.9 years (IQR=3.4-4.0), totalling 1258PY of observation. Of 282 participants who could have reached 48 months of follow-up before censoring, 192 (68%) still used PrEP after 48 months. | | |
| Outcome data | | 15* | | | *Cohort study*—Report numbers of outcome events or summary measures over time | | Results, Sexual behaviour;  Results, Incidence of bacterial sexually transmitted infections;  Results, Incidence rates of HIV and HCV, paragraph 1;  Results, Incidence rates of HIV and HCV, paragraph 2;  Results, Regimen switching;  Results, Discontinuation of PrEP use;  Results, Intracellular TFV-DP concentrations; | | Reported median number of sex partners (past 3 months) was 13 (IQR=6-26), median number of anal sex acts was 18 (IQR=9-43), and median number of CAS acts with casual partners was 10 (IQR=3-20.5) (S1 Table). Numbers of sex partners and anal sex acts decreased with each additional year on PrEP, adjusted for age (adjusted rate ratio [aRR] 0.86/year [95% CI 0.83-0.88] and 0.88/year [95% CI 0.85-0.91], respectively; Table 2).  […]  In 1092 consultations among 289/367 participants, at least one STI was diagnosed during 1258PY: 891 during 914PY among daily PrEP users and 201 during 344PY among event-driven PrEP users (Table 3). IR of any STI was 87/100PY (95%CI=82-92). This was higher for daily PrEP users (97/100PY; 95%CI=91-104) compared to event-driven PrEP users (59/100PY; 95%CI=51-67; p<0.0001; Fig 2; Table 3; S3 Table).  […]  Two daily PrEP users were diagnosed with HIV during follow-up, both in the first year since PrEP initiation, resulting in an IR of 0.2/100PY (95%CI=0.0-0.6; Table 3).  […]  17 incident HCV infections were diagnosed among 15 participants during 1186PY of follow-up (IR=1.4/100PY, 95%CI=0.9-2.3; Table 3). Ten were primary infections and 7 reinfections (S3 Table). Daily PrEP users accounted for 15 HCV infections (IR=1.7/100PY, 95%CI=1.0-2.9) and event-driven PrEP users for two (IR=0.6/100PY, 95%CI=0.2-2.5) (Table 3). IRs of HCV decreased over time: year 1, n=6 (IR=1.8/100PY, 95%CI=0.8-3.9); year 2, n=8 (IR=2.5/100PY, 95%CI=1.3-5.2); year 3, n=2 (IR=0.7/100PY, 95%CI=0.2-2.7); year 4, n=1 (IR=0.4/100PY, 95%CI=0.06-2.6; S5 Table).  […]  Overall, 137/367 (37%) participants switched PrEP regimens 254 times: 141 times from daily PrEP to event-driven PrEP and 113 times from event-driven PrEP to daily PrEP use. The rate of any switch was highest in the first year (26.1/100PY) and decreased over time (IRR 0.87/year [95% CI 0.78-0.98], p=0.019) to 17.2/100PY in the fourth year (S8 Table; Fig 4).  […]  We observed 112 PrEP stops among 98/367 (27%) participants; 31 stops were subsequently followed by a restart. 43 participants had a formal study exit visit and another 43 were lost-to-follow-up. We registered 17 gaps of more than 9 months between study visits, and nine participants reported not having used PrEP for a period of at least three months, despite continuing study participation. Median time until the first stop among those who stopped was 21 (IQR=12-35) months. Overall rate of stopping was 8.3/100PY (95%CI=6.9-10.0). In  […]  Among daily PrEP users, median TFV-DP concentration at 3 or 6 months was 1263 fmol/punch (IQR=1000-1619; n=240), at 12 months 1299 fmol/punch (IQR=1021-1627; n=259), at 24 months 1288 fmol/punch (IQR=1005-1617; n=223) and at 48 months 1693 fmol/punch (IQR=1310-2252; n=127). At 12 months, 93% (n=240/259) of participants had a TFV-DP concentration ≥700 fmol/punch, at 24 months 90% (n=200/223) and at 48 months 94% (n=120/127). | | |
| Main results | | 16 | | | (*a*) Give unadjusted estimates and, if applicable, confounder-adjusted estimates and their precision (eg, 95% confidence interval). Make clear which confounders were adjusted for and why they were included | | Results, Sexual behaviour;  Results, Incidence of bacterial sexually transmitted infections;  Results, Discontinuation of PrEP use  Table 2, Table 3, S2 Table, S3, S5 Table, S6 Table, S7 Table, S9 Table | | Numbers of sex partners and anal sex acts decreased with each additional year on PrEP, adjusted for age (adjusted rate ratio [aRR] 0.86/year [95% CI 0.83-0.88] and 0.88/year [95% CI 0.85-0.91], respectively; Table 2). These changes were also statistically significant when stratified by PrEP regimen (Table 2, Fig 1). Number of CAS acts with casual partners also decreased with each additional year on PrEP, when adjusted for age and testing frequency (aRR 0.92/year [95% CI 0.88-0.97]), also when stratified by regimen (Table 2, Fig 1). Numbers of sex partners, anal sex acts, and CAS acts with casual partners were higher in daily PrEP users compared to event-driven PrEP users (S1 Table).  […]  Compared to the first year, IRs of any STI were lower in the second (aIRR=0.77, 95%CI=0.65-0.91) and third (aIRR=0.78, 95%CI=0.66-0.92) years when adjusted for age and STI testing frequency, also for chlamydia and gonorrhoea (Fig 3; S5 Table). This decrease was not seen in the fourth year (aIRR=0.89, 95%CI=0.75-1.06).  […]  In multivariable analysis, younger age (p=0.036), fewer CAS acts with casual partners (p=0.039), not having a university/university of applied sciences degree (p=0.030) and a MHI-5 score of <60 (p=0.036) were associated with earlier stopping (S9 Table). Being diagnosed with an STI in the past 3 months was not associated with stopping (adjusted HR=0.48, 95%CI=0.14-1.64, p=0.24; S9 Table). | | |
|  |  |  |  |  | (*b*) Report category boundaries when continuous variables were categorized | | Methods, Measures;  Methods, Outcomes, paragraph 4;  Methods, statistical methods, paragraph 3 | | Symptoms of depression or anxiety were assessed using the Mental Health Inventory-5 (MHI-5) score, where a score of <60 indicated symptoms of depression or anxiety. The Alcohol Use Disorders Identification Test (AUDIT) and Drug Use Disorder Identification Test (DUDIT) questionnaires were used to assess problematic alcohol and drug use, respectively; scores ≥8 are interpreted as indicative of alcohol-related or drug-related problems.  […]  We calculated median levels of intracellular tenofovir diphosphate (TFV-DP) in dried blood spots and corresponding IQRs among daily PrEP users and report the proportion of daily PrEP users with good adherence (TFV-DP ≥700 fmol/punch).  […]  To analyse changes in bacterial STI incidence, we calculated STI IRs per 100PY for each 3-monthly follow-up period. We examined whether there was a linear association between time on PrEP and STI incidence using Poisson regression, and thereafter whether STI incidence varied between three-monthly periods within each year of PrEP use, jointly. Because there was a non-linear association between time on PrEP and STI incidence, but no statistical evidence for variation in STI incidence within years (p=0.22), we modelled the change in STI IRs in years 2, 3 and 4 compared to the first year on PrEP. | | |
|  |  |  |  |  | (*c*) If relevant, consider translating estimates of relative risk into absolute risk for a meaningful time period | | n/a | |  | | |
| Other analyses | 17 | | Report other analyses done—eg analyses of subgroups and interactions, and sensitivity analyses | | | | Results, Sexual behaviour, paragraph 2;  Results, Incidence of bacterial sexually transmitted infections | | Results from sensitivity analyses assessing behaviour over time since initiating PrEP while including periods without PrEP use or follow-up were largely the same (S3 Table).  […]  Sensitivity analyses assessing STI incidence since initiating PrEP and ignoring gaps in follow-up yielded comparable results (S6 Table). |  |  |
| Discussion | | | | | | | | | |  |  |
| Key results | 18 | | Summarise key results with reference to study objectives | | | | Discussion, paragraph 1 | | Over the first four years of PrEP use among participants of this prospective demonstration cohort in Amsterdam, the Netherlands, the number of CAS acts with casual partners and the total number of sex partners decreased over time. STI incidence was high, but stable over time. Therefore, these findings do not confirm apprehensions of increasing STIs in the first four years following PrEP initiation. Incidence of HIV was very low and the two incident infections occurred during the first year on PrEP. Objectively measured adherence among daily PrEP users remained well above the protective threshold during study follow-up for the large majority of participants. Retention at 48 months was high (68%). Thus, effectiveness of PrEP continues beyond the previously reported 2-year results in our and other demonstration studies. |  |  |
| Limitations | 19 | | Discuss limitations of the study, taking into account sources of potential bias or imprecision. Discuss both direction and magnitude of any potential bias | | | | Discussion, paragraph 9 | | We acknowledge some limitations. The AMPrEP cohort officially closed at 1 December 2020, but due to COVID-19 measures, we censored data after 15 March 2020, to exclude effects on sexual behaviour and STI incidence resulting from the COVID-19 pandemic. Therefore we were not able to include up to five years of follow-up nor the official end-of-study visits. Second, this cohort presumably included a high proportion of early adopters. Participants were relatively old, and mostly identified as men, white, and were university/university of applied sciences educated and they are unlikely to represent the broader population of MSM and transgender women who could benefit from PrEP. We were only able to include two transgender women. |  |  |
| Interpretation | 20 | | Give a cautious overall interpretation of results considering objectives, limitations, multiplicity of analyses, results from similar studies, and other relevant evidence | | | | Discussion, paragraph 3-9 | | PrEP programme policies select for people who are behaviourally susceptible for HIV and therefore these people are prone to acquire other STIs. As AMPrEP was initiated in 2015, before the European Medicine Agency approved TDF/FTC for PrEP in July 2016, there was no other formal way to acquire PrEP in the Netherlands at the time. The lack in PrEP availability likely resulted in inclusion of a group of early PrEP adopters. After generic PrEP became available in the Netherlands in 2017, a limited but growing number of general practitioners (GPs) started prescribing PrEP. The Dutch national PrEP pilot, offering PrEP at a reduced price and free PrEP-care at public health services to a maximum of 8,500 people, started in July 2019. AMPrEP participants were allowed to exit the study and enter the national PrEP pilot whenever they preferred to. Alternatively, they could be referred to their GP, but PrEP care through GPs in the Netherlands remains insufficiently accessible.  We assessed multiple sexual behaviour measures. We considered the number of CAS acts with casual partners as the most relevant factor in the context of HIV acquisition. The rate of this behaviour decreased over four years of follow-up in both daily PrEP and event-driven PrEP users, with expectedly higher numbers among daily PrEP users. The total number of sex partners also decreased over time, as previously observed by Molina et al. (2022). Grant et al. (2010) noted, in a double blind, randomised, placebo-controlled trial, a reduction of sex partners with whom participants had receptive intercourse over a median follow-up of 1.2 years, and suggested that the services around PrEP use (e.g. counselling) or taking the pill itself could serve as a reminder of HIV risk and contribute to choosing this “safer behaviour”. Reyniers et al. (2021) suggest a broader paradigm of improved sexual health brought about by PrEP, through empowering its users to more actively engage in their sexual health. AMPrEP participants had the opportunity to reflect on their sexual activity with nurses and physicians during each three-monthly consultation. This paradigm of sexual empowerment, leading to more considered sexual decisions, could explain why we observed a reduced numbers of sex partners and condomless anal sex acts with casual partners over time in our cohort. An alternative explanation could be that sexual behaviour changes over time, and participants enrolled in this study when their need for PrEP was particularly high.  AMPrEP and the Be-PrEP-ared project in Belgium were, to the best of our knowledge, the first prospective demonstration projects to offer participants the choice between daily PrEP and event-driven PrEP, including the option to switch between regimens. We observed a high and stable incidence of STIs, similar to other early PrEP studies. We noted this especially among daily PrEP users, as well as higher numbers of sex partners and CAS acts compared to event-driven PrEP users, in agreement with an earlier, pooled analyses of AMPrEP and Be-PrEP-ared over the first 28 months. Daily PrEP appears to be used during periods with more frequent sexual contacts or less condom use, coinciding with an increase in the chance to acquire HIV and STIs. This suggests that PrEP users are capable of deciding which regimen to use, depending on their sexual behaviour.  The low HIV incidence is likely a direct result of high PrEP adherence, as demonstrated by high median levels of TFV-DP around the level of perfect adherence. These levels were also well above the protective thresholds at all time points up to 4 years after PrEP initiation in the majority of participants. A subgroup of participants was included in a nested RCT assessing the effect of an app providing visualised feedback to increase adherence, as reported previously, which could have possibly been a contributing factor to adherence. The absence of any HIV infection after one year on PrEP provides further evidence that PrEP use can be sustainable and efficacious in preventing HIV over the longer course. This finding is in line with other studies with less follow-up time. The stable, high STI rates are comparable to those in contemporaneous studies ranging from 75-98 per 100PY, although differences in methods could explain some of the variation between estimates. Post-exposure prophylaxis using doxycycline can effectively prevent bacterial STIs, and could, in the future, be considered for specific PrEP users. This is currently being adopted in some countries (e.g. the United States), but not in the Netherlands, and long-term effects on antimicrobial resistance remain unknown.  HCV prevalence was high among participants initiating PrEP in AMPrEP as described in previous analyses of our cohort, but during follow-up its incidence decreased over time, parallel to the decrease seen in the Dutch population with HIV since direct-acting antivirals for HCV became widely available in 2015.  In our cohort, a substantial proportion of participants switched PrEP regimens once or multiple times, especially during the first year of PrEP use. The rate of switching from event-driven to daily PrEP was higher than vice versa. Previous qualitative research suggested recurring side-effects at re-initiation of PrEP during event-driven use, and difficulties in adhering to an irregular regimen, as reasons for this shift. Retention to PrEP remained high over four years and the vast majority of the daily PrEP users had high adherence levels at each measurement. The majority of participants that stopped using PrEP, did so because of low self-perceived need for PrEP, as reported previously. However, stopping was also associated with younger age, not having a university/university of applied sciences degree and with having signs of depression/anxiety in our study. Furthermore, there can be discordance between self-perceived and actual need for PrEP. This can leave ex-PrEP users vulnerable to HIV, as supported by reports of high HIV incidence among people who discontinued PrEP, stressing the importance of low-threshold access to PrEP and adherence and persistence counselling so PrEP users may discontinue PrEP well-informed. PrEP providers should make an effort to confirm that those who are lost-to-follow-up are using alternative HIV prevention strategies, or are invited into PrEP care again, if necessary.  A major strength of this study is the long follow-up time of up to four years. In real-world settings, many are likely to use PrEP for several years and very little data were available on long term (i.e. longer than two years) PrEP use. Second, AMPrEP was a prospective, observational cohort, enabling a prospective assessment of outcome measures in which STI diagnoses made in-between study visits were included. Third, AMPrEP was among the first two demonstration projects allowing participants to choose between daily PrEP and event-driven PrEP use, adapt their PrEP use to match their need of protection, allowing an independent and long-term assessment of both regimens and inter-regimen switching behaviour.  We acknowledge some limitations. The AMPrEP cohort officially closed at 1 December 2020, but due to COVID-19 measures, we censored data after 15 March 2020, to exclude effects on sexual behaviour and STI incidence resulting from the COVID-19 pandemic. Therefore we were not able to include up to five years of follow-up nor the official end-of-study visits. Second, this cohort presumably included a high proportion of early adopters. Participants were relatively old, and mostly identified as men, white, and were university/university of applied sciences educated and they are unlikely to represent the broader population of MSM and transgender women who could benefit from PrEP. We were only able to include two transgender women. |  |  |
| Generalisability | 21 | | Discuss the generalisability (external validity) of the study results | | | | Discussion, paragraph 9;  Discussion, paragraph 10 | | Second, this cohort presumably included a high proportion of early adopters. Participants were relatively old, and mostly identified as men, white, and were university/university of applied sciences educated and they are unlikely to represent the broader population of MSM and transgender women who could benefit from PrEP. We were only able to include two transgender women.  […]  Future PrEP studies should aim to include a more representative sample for the population susceptible to HIV. |  |  |
| Other information | | |  | | | | | | |  |  |
| Funding | 22 | | Give the source of funding and the role of the funders for the present study and, if applicable, for the original study on which the present article is based | | | | 9, 21 | | Role of the funders  The study funders had no role in study design, data collection, data analysis, data interpretation, nor in writing of the manuscript.  The AMPrEP study received funding as part of the H-TEAM initiative from ZonMw (grant number: 522002003), the National Institute for Public Health and the Environment (RIVM), GGD research funds and the H-TEAM. The study drug and an unrestricted research grant for AMPrEP was provided by Gilead Sciences. The H-TEAM initiative is supported by the Aidsfonds Netherlands (grant number: 2013169), Stichting Amsterdam-Dinner Foundation, Gilead Sciences Europe Ltd (grant number: PA-HIV-PREP-16-0024), Gilead Sciences (protocol numbers: CONL-276-4222,CO-US-276-1712), Janssen Pharmaceuticals (reference number: PHNL/JAN/0714/0005b/1912fde), M.A.C. AIDS Fund and ViiV Healthcare (PO numbers: 3000268822, 3000747780). |  |  |

*Give information separately for cases and controls in case-control studies and, if applicable, for exposed and unexposed groups in cohort and cross-sectional studies.

**Note:** An Explanation and Elaboration article discusses each checklist item and gives methodological background and published examples of transparent reporting. The STROBE checklist is best used in conjunction with this article (freely available on the Web sites of PLoS Medicine at http://www.plosmedicine.org/, Annals of Internal Medicine at http://www.annals.org/, and Epidemiology at http://www.epidem.com/). Information on the STROBE Initiative is available at www.strobe-statement.org.
