# Supplementary material for: Sexual behaviour and incidence of sexually transmitted infections among men who have sex with men (MSM) using daily and event-driven pre-exposure prophylaxis (PrEP): Four-year follow-up of the Amsterdam PrEP (AMPrEP) demonstration project cohort
Source: PLoS Med. 2024 May 8;21(5):e1004328. doi: 10.1371/journal.pmed.1004328 (PMC11111007; doi:10.1371/journal.pmed.1004328)
Supplement: Attachment — Submitted filename: vandenelshout.pdf [file pmed.1004328.s012.pdf]

This paper describes a follow–up of a cohort in Amsterdam of MSM taking PrEP.

I was asked for a statistical report and I interpret that to include all aspects of the design and conduct of the study.

Points of detail

Page 5 Despite having spoken (British) English all my life I still find bi–annual and biennial confusing. So I looked them up on–line just to be sure. Much to my surprise I find that in US English biannual can mean either every 6 months or every 24 months although that is not true in British English. I think I would avoid it in favour of every 6 or 24 months.

Page 6 What exactly is implied by the phrase 'on a discrete scale'? Perhaps clarify?

Page 8 Why was it felt necessary to categorise age and STI testing frequency? Categorising an essentially continuous variable wastes information (Altman and Royston, 2006; Royston et al., 2006) and leads to models which are often implausible as they predict the effect remaining flat within categories and then jumping to a new value at the category boundary. As far as I can see they are only used in the main analyses as adjustment variables so some smooth function like splines or low–degree polynomials could have been used. For presentation in some of the tables categorising is probably inevitable.

Page 9 I would suggest ending the sentence after 'Cox regression'and then replacing 'which' with 'The factors/variables/covariates'. At the moment it is unclear what the antecedent of 'which' is.

Page 12 Looking at Figure 2 I do not see a clear pattern emerging in contrast to the impression given by the text here. Figure 2 does not seem to support comparing years since the picture within years is so variable. If this is an essential finding then I think some formal test of the overall shape of the relationship would be indicated.

Page 16 Translating between national education systems is always tricky. I take it that college or university is intended to cover any post 18 education whether it leads to a degree or not. If I am wrong that needs clarifying.

Figure 4 The main interest here is in the transitions between states but the

transitions from blue to red and vice versa are very hard to see. Reducing the saturation of the green would help here I think. I would also try replacing green with pale yellow as people with the most common colour vision anomaly are going to struggle with red lines on a green background.

Supplementary Table 1 Perhaps state where the p–values come from? I was surprised to see the 1.00 values which I cannot replicate with Fisher's exact test (although I do not know how many are missing in each group). I am not saying what has been done is wrong but it would save someone else from being surprised. Most (but not all) of the other tables have this information.

Summary

Mostly requests for clarification.

Michael Dewey

References

D G Altman and P Royston. The cost of dichotomising continuous variables. *British Medical Journal*, 332:1080, 2006.

P Royston, D G Altman, and W Sauerbrei. Dichotomizing continuous predictors in multiple regression: a bad idea. *Statistics in Medicine*, 25:127–141, 2006.
